# Research in the “Games Bubble”: leveraging outreach-embedded data collection at the 2025 Harbin Asian Winter Games

**DOI:** 10.3389/fspor.2026.1945134

**Published:** 2026-10-07

**Authors:** Gobinathan Nair, Andrea Petróczi, Xie Li, Ge Yifan, Venera Abdulla, Nadiah AL-Shamali

**Affiliations:** 1 Southeast Asia Regional Anti-Doping Organization, Singapore, Singapore; 2 School of Life Sciences, Pharmacy and Chemistry, Faculty of Health, Science, Social Care and Education, Kingston University, Kingston upon Thames, United Kingdom; 3 Faculty of Health and Sport Sciences, Széchenyi István University, Győr, Hungary; 4 Communications and Education Department, China Anti-Doping Agency, Beijing, China; 5 Regional Anti-Doping Organization of Central Asia, Almaty, Kazakhstan; 6 Olympic Council of Asia, Hawalli, Kuwait

**Keywords:** anti-doping, athlete, awareness, clean sport, legitimacy, morality, outreach, questionnaire

## Abstract

**Introduction:**

Anti-doping social science has been shaped largely by Western and Eurocentric evidence, with limited research capturing athlete perspectives from geographically heterogeneous and potentially culturally diverse contexts. Major multisport events provide an opportunity to address this gap, especially when research is embedded within athlete outreach. This study examined the feasibility and value of outreach-embedded research aligned with values-based anti-doping outside traditional research settings.

**Methods:**

Data were collected during athlete outreach activities at the 2025 Asian Winter Games (Harbin, China) using a brief online questionnaire completed by athletes (*n* = 293), coaches (*n* = 27), team officials (*n* = 39), medical personnel (*n* = 22), other athlete support personnel (*n* = 18), and athlete ambassadors/engagement personnel (*n* = 3), representing 28 countries and regions. Survey items assessed motivations for engaging with outreach, satisfaction and perceived impact, and “values in action,” including meanings and importance of clean sport, moral evaluations of performance-enhancement practices, and perceptions of anti-doping legitimacy.

**Results:**

A total of 402 participants from 28 countries completed the outreach-embedded survey. Engagement with the Athlete Engagement and Education Centre was highly positive: satisfaction scores across content, activity format, and engagement averaged 4.74–4.78/5, 87% of respondents reported being very satisfied, and the program received a mean overall rating of 9.26/10. More than 72% strongly agreed that the outreach increased anti-doping awareness and knowledge and contributed positively to their Games experience, with no significant differences across athletes, athlete support personnel, and officials. Participants also reported high importance of clean sport and strong support for the legitimacy of the current anti-doping system. At the same time, responses showed moral nuance: 69.2% viewed deliberate use of prohibited substances as morally unacceptable under any circumstances, whereas others endorsed conditional acceptance, and legally permissible or medically justified enhancement practices were more widely accepted.

**Conclusion:**

Outreach and survey content created a productive synergy, generating both positive participant experience and valuable data. Integrating outreach and research at regional multinational multisport events can offer a practical route to diversify the anti-doping evidence base while helping to catalyze deeper, locally grounded research agendas over time.

## 1 Introduction

Social science research on anti-doping has expanded considerably over the past two decades; however, its empirical foundations remain uneven (1–4). Much of the existing evidence continues to be derived from athletes and athlete support personnel (ASP) located in Europe, North America, and other economically developed sport systems (5). This dominance of a largely Eurocentric epistemology has shaped prevailing assumptions about clean sport, moral reasoning, and perceptions of anti-doping efforts, often implicitly treating these perspectives as universal (6, 7). As a result, cultural, developmental, and sport-specific diversity in how anti-doping is understood and experienced remains insufficiently captured within mainstream research (5, 8).

This imbalance is not merely geographic. It reflects deeper asymmetries in whose experiences, values, and moral frameworks inform knowledge production in anti-doping. Athletes from under-represented regions—including much of Asia, Africa, and parts of Latin America—often train and compete under materially different conditions, within distinct sporting microcultures, and in regulatory environments shaped by diverse historical relationships with authority, fairness, and institutional trust. Yet these contextual differences are rarely examined empirically at scale, limiting the field's capacity to determine—on the basis of evidence rather than assumption—whether culturally responsive education, policy, and engagement strategies are required. From an epistemic standpoint, athletes are not merely subjects of regulation or objects of education, but knowledge holders whose perspectives are essential for understanding clean sport in a global context.

### 1.1 The “Games Bubble”: a naturalistic research environment

The relative absence of research from regions beyond Europe and other highly resourced sport systems likely reflects a combination of structural and practical constraints, including limited access to research infrastructure and expertise, logistical and language barriers to reaching elite athlete populations, and resource constraints affecting data collection (3, 9, 10). Major multinational, multisport events offer a unique opportunity to address these gaps. Such events bring together athletes and ASP from diverse geographical regions, cultural and religious traditions, developmental sport systems, and sport-specific microcultures within a shared regulatory environment. When combined with structured outreach activities, they create rare conditions for collecting data that are both geographically heterogeneous and potentially culturally diverse, and ecologically valid.

Major sport events have long been recognised as valuable naturalistic environments for studying athletes' experiences, behaviours, and knowledge. The temporary co-location of diverse athlete populations, combined with the unique pressures and cultural dynamics of competition, creates a setting in which participants who are otherwise geographically dispersed and difficult to access can be reached. A growing body of literature demonstrates that data collection at major sport events is both feasible and methodologically productive, particularly when using surveys and interviews administered on site.

Empirical studies support this position. Kristiansen and Roberts (11), for example, demonstrated that qualitative interviews can be successfully embedded within Olympic-style events to capture athletes' lived experiences. Similarly, MacIntosh and colleagues (12–14) examined athlete satisfaction and service environments at the Commonwealth Games, highlighting how event context shapes both experience and performance. Within anti-doping, Königstein et al. (15) showed that large-scale survey research is feasible even in high-performance settings, while Gatterer et al. (16) highlighted the importance of context-sensitive, interactive educational delivery. Complementary work with para-athletes (17) reinforces the need to capture diverse perspectives to inform policy and practice.

### 1.2 Outreach at major events: from education to engagement

In parallel with these research developments, outreach activities, particularly in anti-doping, have become increasingly common at major sport events. Event-based education is now an integral component of WADA's International Standard for Education (ISE) (18), reflecting the importance of providing timely, accessible, and context-specific learning opportunities for athletes and athlete support personnel. WADA's outreach model has been in place for more than two decades and has supported anti-doping organisations (ADOs) in establishing and delivering outreach booths at sporting events. This model should be distinguished from WADA-led outreach booths, which are managed and delivered directly by WADA and have, over time, evolved into broader athlete engagement activities. Despite differences in format, ownership, and delivery, both outreach and athlete engagement initiatives share similar aims: to raise awareness, promote clean sport values, increase athletes' access to reliable anti-doping information, and create opportunities for dialogue within the sporting environment.

Evidence suggests that such initiatives are effective when embedded within event environments. For example, Fagnani et al. (19) demonstrated that outreach can meaningfully engage young athletes, while Mountjoy et al. (20, 21) showed that embedding surveys within safeguarding outreach at Youth Olympic Games generated valuable insights alongside high participation rates. Further evidence from other event contexts supports this conclusion. Hong et al. (22) reported strong engagement among Pacific Island athletes participating in research embedded within educational programming at the 2019 Pacific Games.

Collectively, these studies indicate that athletes are willing to engage with research when it is situated within familiar, supportive, and meaningful contexts. Yet, despite these advances, research and outreach have largely remained conceptually and operationally separate. Research is typically framed as data extraction, whereas outreach is treated as education or communication. What remains underdeveloped is an integrated model in which data collection itself becomes a mechanism for engagement.

### 1.3 Integrating outreach and research

An integrated approach would position research instruments—such as surveys or structured interviews—not only as tools for data collection but also as prompts for reflection, dialogue, and learning. Such an approach allows researchers to identify knowledge gaps in real time and address them through immediate interaction with athletes and ASP. This model aligns with evidence that athletes respond positively to interactive, discussion-based educational formats embedded within their competitive environments (16, 20–22). When framed as part of a supportive outreach process rather than as a “bolted-on” function, data collection becomes a mutually beneficial activity, enhancing engagement, strengthening educational impact, and generating richer, more contextually grounded data.

Importantly, integrating research with outreach also addresses limitations of current practice. Outreach initiatives often lack systematic evaluation, limiting their ability to adapt and improve. At the same time, concerns that athletes may perceive surveys as burdensome appear overstated. Evidence suggests that athletes are willing to participate, particularly when activities are voluntary, contextually relevant, and embedded within informal and socially engaging environments. By reframing data collection as collaborative and interactive, this approach enhances both methodological value and athlete experience.

### 1.4 Aims

The present study examines the feasibility and value of integrating research within anti-doping outreach activities at a major regional multisport event: the 2025 Asian Winter Games in Harbin, China. Using a brief survey administered as part of athlete outreach, the study explores both the practical potential of outreach-embedded data collection and the substantive insights such an approach can generate in a geographically heterogeneous and potentially culturally diverse event setting. Guided by the International Standard for Education (18) and the conceptual framework of *values in action* (Figure 1), the study investigates how anti-doping-related values are expressed through participants' interpretations of clean sport, the importance they attach to clean sport, their moral evaluations of performance-enhancement practices, and their perceptions of anti-doping legitimacy. In parallel, the study examines how participants engage with and evaluate the outreach activity itself.

**Figure 1:**
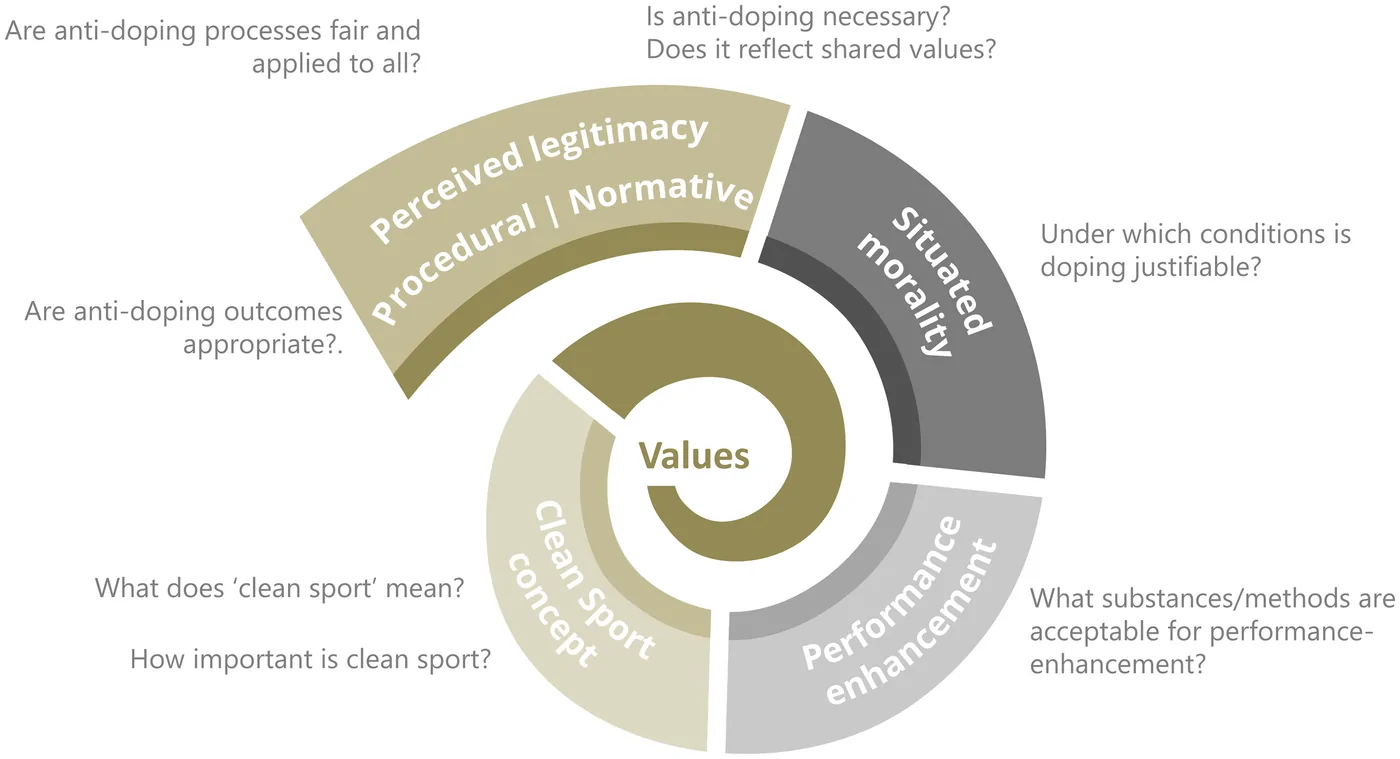
Conceptual framework illustrating how values are expressed in action through clean sport meanings, moral evaluations of performance enhancement, and situated morality, and how these inform perceptions of normative and procedural anti-doping legitimacy.

Accordingly, the study addresses the following research questions: (1) *How was the outreach activity, including the embedded survey, experienced and evaluated by athletes, athlete support personnel (ASP) and officials?;* (2) *How do athletes, ASPs and officials participating in the Games interpret and value clean sport?;* (3) *How are different performance-enhancement practices morally evaluated in this setting?;* and (4) *How is anti-doping legitimacy perceived in this event context?*

## 2 Methods

With the event setting addressing the *where* of data collection, construct selection addressed the complementary question of *what* to measure. The integration of outreach, education, and research requires a close alignment between educational content and the research constructs used to capture participants' perspectives. The values-based approach mandated by the International Standard for Education (ISE) provided the foundation for this alignment. The ISE, first introduced under the 2021 World Anti-Doping Code, established values-based education as a foundational component of anti-doping education, and the revised 2027 ISE retains this emphasis as a mandatory element of education programmes. Specifically, the 2027 ISE positions education as a core component of prevention, highlights the preservation of the spirit of sport and the fostering of a clean sport culture as its overarching purpose, and explicitly advocates educating wider populations through values-based education to instil clean sport values and reinforce clean sport behaviours.

### 2.1 Conceptual underpinning

Values sit at the core of anti-doping education and underpin several key constructs of interest in social science research, including moral reasoning, perceptions of fairness, and legitimacy. This creates a strong conceptual basis for outreach-embedded research designed to capture how values are expressed in action. In the values-based framework guiding this study (Figure 1), values are not treated as abstract orientations, but as enacted through the meanings attached to clean sport (Petróczi et al., 23), the importance attributed to clean sport (6), concrete judgements about acceptable and unacceptable performance-enhancement practices (6, 24), and perceptions of anti-doping legitimacy (25). Within this framework, normative legitimacy is reflected in how athletes interpret and value clean sport and in how they morally evaluate enhancement practices. Procedural legitimacy, by contrast, is shaped by lived and observed experiences of testing, education, rule implementation, and enforcement. Although expectations of fairness are inherently value-laden, they influence procedural legitimacy only insofar as they are confirmed or challenged in practice. Empirical research suggests that negative procedural experiences can undermine trust and engagement, even when athletes broadly endorse clean sport values (26–28). Related work also indicates that compliance is often rooted in respect towards authority, rules, or both (29). Legitimacy is therefore not simply an abstract belief, but something constructed through lived experience, making context and culture central to understanding how athletes interpret and respond to anti-doping systems.

Despite growing attention to these dynamics, research examining moral reasoning and legitimacy outside Eurocentric settings remains limited. This is a significant blind spot for a global regulatory system that is applied across a geographically, institutionally and culturally diverse sport populations. The present study therefore used a values-based framework to guide construct selection, enabling the embedded survey to align with the educational purpose of outreach while also capturing participants' interpretations of clean sport, moral evaluations of enhancement practices, and perceptions of anti-doping legitimacy.

### 2.2 Study context: Asian Winter Games 2025, Harbin

Data were collected during the Asian Winter Games 2025, held in Harbin, China, as part of the official Athlete Engagement and Outreach activities delivered alongside the event. The Asian Winter Games represent a major multisport, multinational competition, bringing together athletes from a wide range of geographical regions, cultural traditions, religious backgrounds, and sport-specific microcultures within a single regulatory environment. As such, the Games provided an ecologically valid setting for examining athletes' perceptions of clean sport, anti-doping engagement, and institutional legitimacy.

Crucially, data collection was embedded within the broader outreach program rather than conducted as a separate research activity. Two booths were established for this purpose: the China Anti-Doping Agency's (CHINADA) Clean Sport Outreach booth and the Olympic Council of Asia (OCA) Anti-Doping booth, which formed part of OCA's Guarding the Games program. Participants engaged in interactive activities such as games and knowledge quizzes with souvenir items given upon completion. This design aligns with the study's epistemic aim of capturing perceptions as they are formed and expressed *in situ*, during moments of interaction with the anti-doping system, rather than as retrospective or hypothetical judgements. Positioning research within outreach activities also reflects an intentionally non-extractive approach, where engagement, dialogue, and feedback generation are integral to both practice and knowledge production.

### 2.3 Study design

The study employed a cross-sectional survey design implemented within the Athlete Engagement framework at the Games. A short, online questionnaire was used to minimise participant burden and maximise accessibility across linguistic, cultural, and sport–specific contexts. The survey was administered at both CHINADA's and OCA's outreach booths, where athletes, ASPs and other visitors (attending the Games in official capacity) were invited to participate as part of the outreach activities. Participation was voluntary and unrelated to any testing, monitoring, or compliance procedures.

The questionnaire was designed to serve a dual function. First, it collected practice-relevant feedback on outreach activities, supporting iterative improvement of engagement strategies. Second, it generated research data on athletes' motivations for engagement, satisfaction with outreach, and broader perceptions relevant to legitimacy and clean sport. This dual-purpose design reflects the study's core premise that outreach and research need not operate as separate or competing activities, but can be mutually reinforcing when carefully aligned.

The questionnaire was available in English and Chinese. Both language versions were drawn from the pre-existing WADA outreach survey. The Chinese version had previously been developed through translation and back-translation by anti-doping personnel with bilingual capabilities and used at previous outreach events. The language version selected by individual participants was not retained in the research dataset.

### 2.4 Participants

Participants were athletes competing at the Asian Winter Games 2025, along with athlete support personnel and officials attending the Games, who voluntarily engaged with the Outreach booths during the data collection period. No incentives were offered beyond what was part of the athlete engagement for completion of games and knowledge quizzes, and no eligibility restrictions were imposed beyond Games accreditation as an athlete. Specifically, participants received souvenirs for completing the outreach activities. Completing the questionnaire was not a condition to receive souvenirs. The inclusive recruitment approach was intended to capture a broad range of sporting disciplines, national affiliations, and experiential backgrounds.

Demographic variables were purposefully kept limited to reduce intrusiveness and support engagement within a time-constrained event setting. The emphasis was placed on capturing experiential and perceptual data rather than creating a demographically exhaustive profile, consistent with the study's focus on legitimacy perceptions and situated moral reasoning.

### 2.5 Procedures

Data collection took place between February 6 and 13, 2025. Participants were invited in person by trained Clean Sport Ambassadors, who were part of the outreach delivery team. Access to the questionnaire was hosted online and accessed via mobile phone or tablet, either through a general mobile submission link or WeChat.

Clean Sport Ambassadors invited participants to complete a questionnaire designed to gather feedback on the outreach activities and explore perspectives on clean sport and anti-doping. The peer-led engagement process involved an ambassador invitation, brief verbal orientation, participation in interactive activities, and an optional survey, with a souvenir provided following activity completion. This approach was intended to minimize perceived power differentials and promote engagement within a collaborative rather than supervisory environment.

All study information was provided verbally by the Clean Sport Ambassadors. Participants were informed that survey participation was voluntary, responses were anonymous, and data would be used solely for research and program development purposes. They were also assured that participation would not affect their involvement in the Games or their relationship with anti-doping authorities. Principles of voluntary participation, confidentiality, and respect for participants were upheld throughout.

### 2.6 Measures

The outreach-embedded questionnaire comprised 22 questions capturing participant context, outreach engagement, and perceptions relevant to clean sport, situated moral reasoning, and anti-doping legitimacy. Questions were designed for rapid completion in a major event setting, thus they were set in closed-response format with single or multiple selection options (see Supplementary File S1: Table S1).

#### 2.6.1 Satisfaction and feedback

##### 2.6.1.1 Outreach engagement

Outreach engagement was measured by participants' motivations for visiting the Athlete Engagement & Education Centre, satisfaction with outreach content, and staff interactions, perceived impact on awareness and knowledge, overall program evaluation, and likelihood of recommending outreach to others. Together, these measures captured experiential dimensions relevant to procedural legitimacy, including perceived respect, usefulness, and value of engagement.

##### 2.6.1.2 Outreach activity preference

Participants were asked to select the most enjoyed activities offered at the Outreach centres during the game (see Supplementary File S2). Multiple selection was possible.

#### 2.6.2 Research variables

Values-in-action framework allows for a direct and empirically grounded examination of how moral reasoning, clean sport meanings, and legitimacy perceptions cohere in practice, without assuming that values operate uniformly or independently of context. Consistent with this approach, research questionnaire items were designed to capture how personal values manifest through concrete interpretations, moral judgements, and evaluations, rather than through abstract value endorsement. Responses were recorded on Likert-type scales.

##### 2.6.2.1 Meaning and importance of clean sport

Values were operationalised most directly through participants' conceptualisation of clean sport by selecting between pre-set options (WADA Outreach survey, Youth Winter Olympics, 2020) and the importance they attribute to clean sport. Together, these items reflect how values such as fairness, honesty, and integrity are understood and prioritised in practice.

##### 2.6.2.2 Perceived anti-doping legitimacy

Four items collectively capture anti-doping legitimacy (25). Normative legitimacy was understood as the shared values based on which anti-doping is justified. By contrast, procedural legitimacy is captured through participants' perceptions of whether anti-doping rules are, fair, effective, and equally implemented. While values such as fairness or equity shape expectations, procedural legitimacy judgements are informed primarily by personal experiences, observations, and knowledge of how the system operates, rather than by value endorsement alone. As such, the legitimacy question allowed differentiation between value alignment with anti-doping goals (normative legitimacy) and evaluations of institutional performance (procedural legitimacy).

##### 2.6.2.3 Morality

Morality regarding anti-doping was assessed with a question from WADA Social Science Research Pack (30). This question assessed situated moral reasoning, requiring respondents to distinguish what they find morally acceptable, conditionally acceptable, or unacceptable in specific contexts, and therefore capturing context-dependent distinctions rather than binary rule acceptance.

##### 2.6.2.4 Acceptable means for performance enhancement

Participants were presented with a range of performance-enhancement related practices as asked to identify which one(s) were acceptable to them. The options covered a range of non-prohibited options (e.g., using supplements, over-the-counter medication) and prohibited practices (e.g., using prohibited substances for recovery, training only or in competition).

#### 2.6.3 Demographics

Participant context was recorded through items on country/region, gender, age, role (athlete or athlete support personnel or officials), and sport and discipline. These variables were used to describe the cultural and sport-specific diversity of the sample rather than for inferential subgroup analyses.

Prior engagement with the anti-doping system was assessed through items addressing exposure to anti-doping education before the Games, educational formats (e.g., seminars, e-learning), and education providers (e.g., WADA, National Anti-Doping Organizations). These items contextualized participants' baseline familiarity with anti-doping regulation.

### 2.7 Data analysis

Analysis was conducted in two stages. First, descriptive statistics were generated to summarize overall patterns of engagement, satisfaction, and promoter scores across participants. Correlational analysis focused on how athletes' engagement experiences reflect the perceived importance of clean sport. For multiple-response questions, each option was coded separately as selected or not selected. Option-specific endorsement was compared across athletes, athlete support personnel, and officials using 3 × 2 Pearson chi-square tests of independence, with exact tests used where expected cell frequencies were insufficient. Holm adjustment was applied across the option-level comparisons for each question, and Cramer's V was reported as the effect-size estimate. This approach retained participants as the unit of analysis while accommodating the multiple-response format. Group comparisons between athletes and ASPs were conducted using nonparametric tests (Mann–Whitney test for gender difference in the importance of clean sport and legitimacy perceptions, and Kruskal–Wallis tests for participant groups). Relationships between the value-based concepts and importance of clean sport were expressed with Spearman r. For categorical proportions, 95% Wilson confidence intervals were reported in the supplementary analyses; approximate 95% confidence intervals for Spearman correlations were calculated using Fisher's z transformation. Level of significance was set to *p* < 0.05.

## 3 Results

Of the 402 completed questionnaires, 260 (64.7%) were recorded as general mobile submissions and 142 (35.3%) as submissions through WeChat. Participants completed the questionnaire in less than five minutes (M = 3.23; SD = 2.15; Mdn = 2.83 [IQR = 2.22], range 0.70–26.02 min). A 5% trimmed mean (removing the fastest and slowest 5%) was M = 3.03 min (SD = 1.33) (*n* = 362), indicating the central tendency was similar after down-weighting extreme times.

### 3.1 Participants

A total of 402 participants completed the outreach-embedded questionnaire during the Asian Winter Games 2025. Respondents represented 28 countries, with the largest proportions from China (28.6%), Hong Kong China (10.2%), Kazakhstan (10.2%), India (8.21%), Thailand (6.97%), Japan (5.72%), and Chinese Taipei (5.72%).

Participants were predominantly athletes (72.9%), with the remainder comprising coaches (6.7%), team officials (9.7%), medical personnel (5.5%), other ASP (4.5%) and athletes ambassadors and engagement (0.7%). For analysis, three participant groups were created based on their distinct roles: athletes (*n* = 293, 72.9%), ASP (*n* = 67, 16.7%) and Officials (*n* = 42, 10.4%). Athletes from a wide range of winter sports disciplines took part, with ice hockey (29.7%), curling (19.1%), speed skating (16.4%), and figure skating (9.6%) most strongly represented.

Gender distribution was broadly balanced (athletes: 53.2% male; ASP: 52.2% male, Officials: 52.4% male). The average participant age was M = 25.9 years (SD = 9.5) for athletes, M = 38.1 years (SD = 11.2) for ASP and M = 38.3 (SD = 9.5) for Officials.

Most participants (93.8%) reported having received some form of anti-doping education prior to attending the Games. Among those who had received education, the most commonly reported formats were e-learning courses (67.9%) and seminars (50.7%), indicating that formal, structured education was more frequently reported than self-directed use of outreach leaflets or informal discussion. Education was most often attributed to WADA and/or National Anti-Doping Organizations (NADOs), although this should be interpreted as perceived education provider rather than confirmed delivery responsibility. Notably, among athletes, 146 of 293 valid cases (49.8%) identified WADA either alone or in combination with another provider, while NADO was also frequently selected. However, the data do not allow a reliable estimate of the percentage of athletes who specifically used WADA-developed resources, such as ADEL, when these were implemented through NADOs. Because WADA provided education solutions can be delivered, contextualized, or administered by NADOs, responses coded as NADO may still include WADA-developed content, while responses coded as WADA may reflect participants' recognition of the source rather than the direct provider. A further notable pattern was the overlap in provider attribution, particularly among athletes: WADA and NADOs were reported at similar levels, and WADA was frequently selected in combination with other organizations. This suggests that participants may experience anti-doping education as a multi-provider ecosystem and may not reliably distinguish between WADA-developed education resources and education delivered or implemented by NADOs.

Despite the high-level of prior exposure, athletes and ASPs actively engaged with outreach activities at Harbin 2025 for multiple reasons. Among athletes, 94.2% had anti-doping education, and 61.1% were tested for doping in the 12-month period leading up to the Games. Most of those were tested between 1 and 5 times (71.5%), followed by 6–10 times (21.3%), 11–15 times (5.0%) and more than 15 times (2%).

### 3.2 Engagement with anti-doping outreach

The most frequently cited motivations for visiting the Athlete Engagement & Education Centre were team requirements (36.3%), a desire for more information about anti-doping (27.6%), peer recommendation (21.9%), and spontaneous interest while passing by the booth (23.1%). Notably, nearly one in five participants reported having visited similar outreach booths at previous major events and choosing to engage again, suggesting cumulative value rather than outreach fatigue. Preferences for outreach activities are shown in Table 1.

**Table 1:** Preference for athlete engagement activities at the Harbin Winter Games (percentage is calculated from all votes within the group).

Activity	Athletes	ASP	Officials
Education exhibition (including ADRV, the Prohibited List, brief doping control procedure)	81 (13.8%)	19 (14.7%)	12 (15.6%)
Interactive games—Frisbee Throwing	60 (10.2%)	15 (11.6%)	8 (10.4%)
Interactive games—Ring Toss to the Spirit of Sport	48 (8.2%)	12 (9.3%)	5 (6.5%)
Interactive games—Snow Run	101 (17.2%)	25 (19.4%)	11 (14.3%)
Interactive games—The Prohibited Substances Shooter	29 (4.9%)	12 (9.3%)	0
Quiz game	134 (22.8%)	24 (18.6%)	21 (27.3%)
Signature and photo	135 (23.0%)	22 (17.1%)	20 (26.0%)

Overall satisfaction with the outreach activities was high. Across content, activity format, and staff/volunteer engagement, average satisfaction scores were 4.74–4.78 out of 5, with 87% of respondents reporting being “very satisfied.” Participants also rated the overall athlete engagement program highly (mean score 9.26 out of 10) and expressed strong willingness to recommend the outreach center to others, with almost 80% indicating they were likely or extremely likely to do so.

The most highly rated engagement activities across the three participant groups were the “Signature & photo” activity and the WADA's anti-doping knowledge quiz. Among athletes, the two most frequently selected activities were Signature & photo (135 votes; 23.0%) and the quiz game (134 votes; 22.8%), followed by Snow Run (101 votes; 17.2%). A similar pattern was observed among officials, for whom the quiz game was the most frequently selected activity (21 votes; 27.3%), closely followed by Signature & photo (20 votes; 26.0%). Among athlete support personnel (ASP), preferences were more evenly distributed, although the Snow Run activity received the highest proportion of selections (25 votes; 19.4%), followed by the quiz game (24 votes; 18.6%) and Signature & photo (22 votes; 17.1%).

More traditional educational activities, including the education exhibition, attracted a moderate level of engagement across all groups, accounting for approximately 14%–16% of selections. By contrast, some of the more game-based activities, particularly The Prohibited Substances Shooter, received fewer selections overall and no votes from officials. Because participants could select multiple activities, the number of recorded “votes” does not correspond to the number of participants within each role category.

### 3.3 Perceived impact of outreach engagement

Participants overwhelmingly reported that visiting the Athlete Engagement & Education Centre had a positive impact on their Games experience. More than 72% strongly agreed that outreach engagement contributed positively to their overall event experience, increased their awareness of anti-doping issues, and enhanced their anti-doping knowledge. These positive evaluations were consistent across participant groups (Table 2).

**Table 2:** Perceived impact of outreach engagement across participant groups.

Impact	Athlete	ASP	Officials	All
Visiting the Harbin 2025 Athlete Engagement and Education Centre increased my awareness of anti-doping issues.	1.59 (0.82)	1.72 (0.62)	1.62 (0.91)	1.61 (0.80)
Visiting the Harbin 2025 Athlete Engagement and Education Centre increased my knowledge of anti-doping.	1.60 (0.86)	1.70 (0.63)	1.60 (0.91)	1.61 (0.83)
Visiting the Harbin 2025 Athlete Engagement and Education Centre contributed actively to my Harbin 2025 experience.	1.56 (0.88)	1.72 (0.69)	1.50 (0.99)	1.58 (0.69)
Please give an overall score of the Harbin 2025 Athlete Engagement Program (scored from 1 to 10)	9.20 (1.48)	9.40 (1.37)	9.45 (1.17)	9.26 (1.43)

Presented as means (SD), scored as −2 (strongly disagree) to +2 (strongly agree).

Nonparametric tests (Kruskal–Wallis) revealed no significant differences between athletes, ASP, and officials in perceived contribution to the Games experience [*H*(2) = 2.19, *p* = .334], increased awareness [*H*(2) = 1.40, *p* = .497], perceived increase in knowledge [*H*(2) = 0.31, *p* = .857], or overall programme evaluation [*H*(2) = 2.71, *p* = .258]. These findings indicate that outreach activity was not perceived merely as a compliance requirement or information provision exercise, but as a meaningful and valued component of the Games environment across all participant roles.

Correlation analyses indicated strong positive associations among participants' evaluations of the outreach activity. Overall experience was highly correlated with both reported increases in awareness (*r* = .866, *p* < .01) and knowledge (*r* = .884, *p* < .01), suggesting that these components formed a closely integrated perception of the outreach programme. Moderate positive correlations were also observed between overall experience and overall evaluation (*r* = .453, *p* < .01), indicating that more positive experiential engagement was associated with more favourable overall judgements of the outreach. Similarly, increases in awareness and knowledge were very strongly interrelated (*r* = .903, *p* < .01) and moderately associated with overall evaluation (*r* = .478 and.468, respectively; *p* < .01). Importantly, the perceived importance of clean sport showed small but statistically significant positive correlations with all outreach-related variables (*r* = .246–.287, *p* < .01). This pattern suggests that participants who placed greater importance on clean sport tended to report slightly more positive engagement with, and perceived benefits from, the outreach activity.

### 3.4 Meanings and importance of clean sport

The overall sample mean was 1.43 (SD = 1.22) on a −2 to +2 scale, suggesting fairly high importance participants placed in clean sport. Ratings of the importance of clean sport were broadly similar across participant groups. Overall, athletes reported a mean score of 1.36 (SD = 1.26), compared with 1.66 for athlete support personnel (ASP; SD = 0.99) and 1.50 for officials (SD = 1.22). Given the ordinal nature of the scale, group differences were examined using a Kruskal–Wallis test. The results indicated that differences between groups did not reach statistical significance, *H*(2) = 5.75, *p* = .057 (exact *p* = .056). Although ASP and officials reported numerically higher importance ratings than athletes, the effect was small (*ε*^2^ = .014) and the KW-H value fell just short of conventional significance thresholds. These findings suggest a high and largely shared endorsement of clean sport across roles, with only limited variation in the degree of importance attached to it.

As shown in Figure 2, female athletes attached greater importance to clean sport than male athletes (*U* = 8,127.0, *p* < .001), whereas no gender difference was observed among ASP (*U* = 551.0, *p* = .868. Although female officials also reported higher ratings than male officials (*U* = 158.5, *p* = .029), this difference did not remain significant after adjustment for the three gender comparisons (see Supplementary Material 3, Table S3.1).

**Figure 2:**
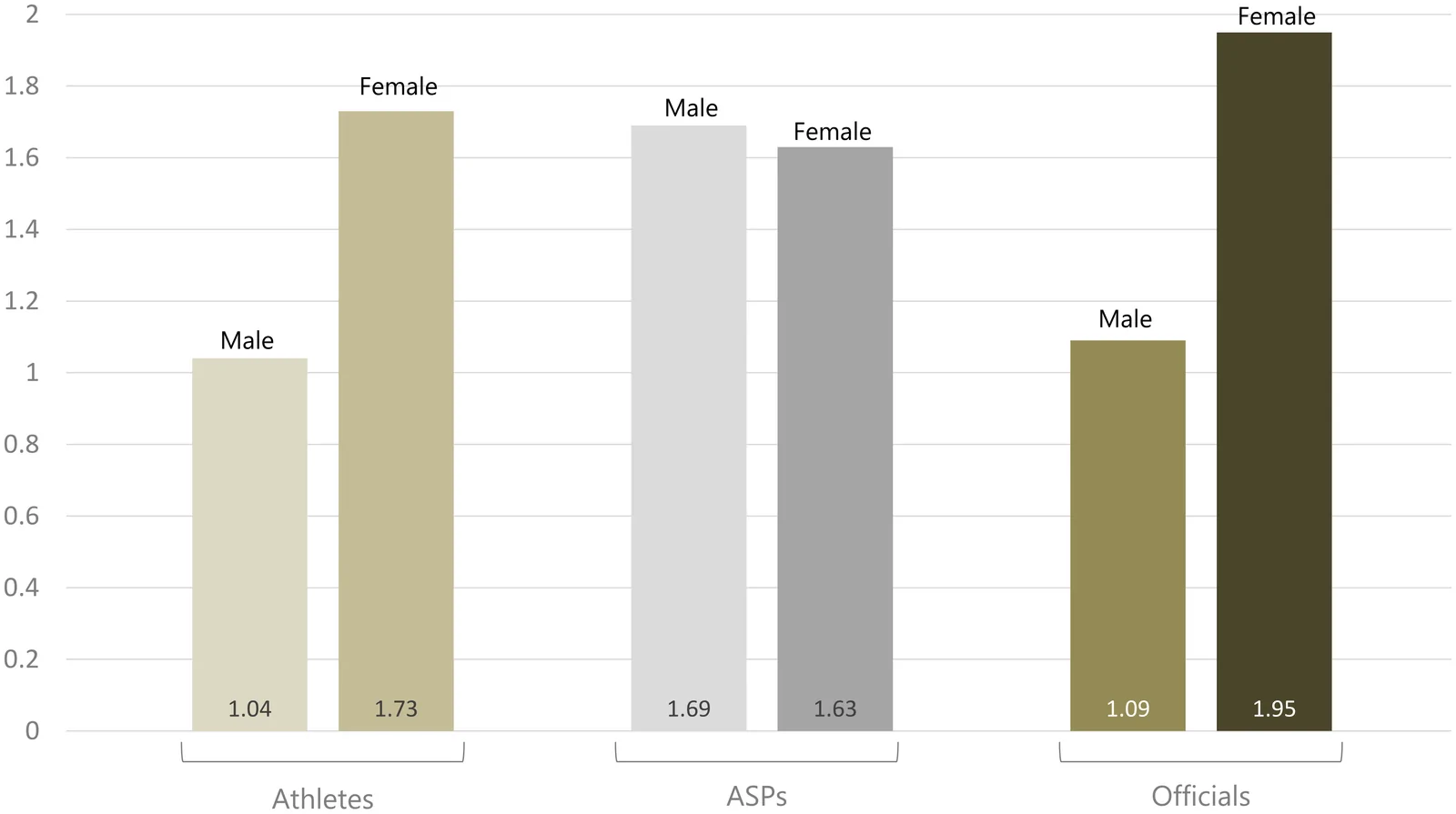
Importance attached to clean sport across athletes, athlete support personnel (ASP), and officials, and reported gender.

Endorsement of different meanings of clean sport is shown in Figure 3. When asked what “clean sport” meant to them, more participants associated the concept with “substances” [i.e., not using prohibited substances and/or methods (69.4%)] than “rules” [i.e., not cheating (51.7%)]. Because participants could select more than one meaning of clean sport, each option was recoded as a binary selected/not-selected variable and compared across athletes, ASP, and officials using separate 3 × 2 contingency-table tests. Descriptively, “not using prohibited substances and/or methods” and “not cheating” were the most frequently endorsed meanings across groups. The only unadjusted role-based difference was observed for “not cheating,” *χ*^2^(2) = 8.28, *p* = .016, although this did not remain statistically significant after Holm correction for multiple comparisons, adjusted *p* = .080. See Supplementary File S3: Table S3.2. Overall, the findings therefore suggest broadly similar patterns of endorsement across participant roles, with some descriptive variation in the relative emphasis placed on rule-based meanings of clean sport.

**Figure 3:**
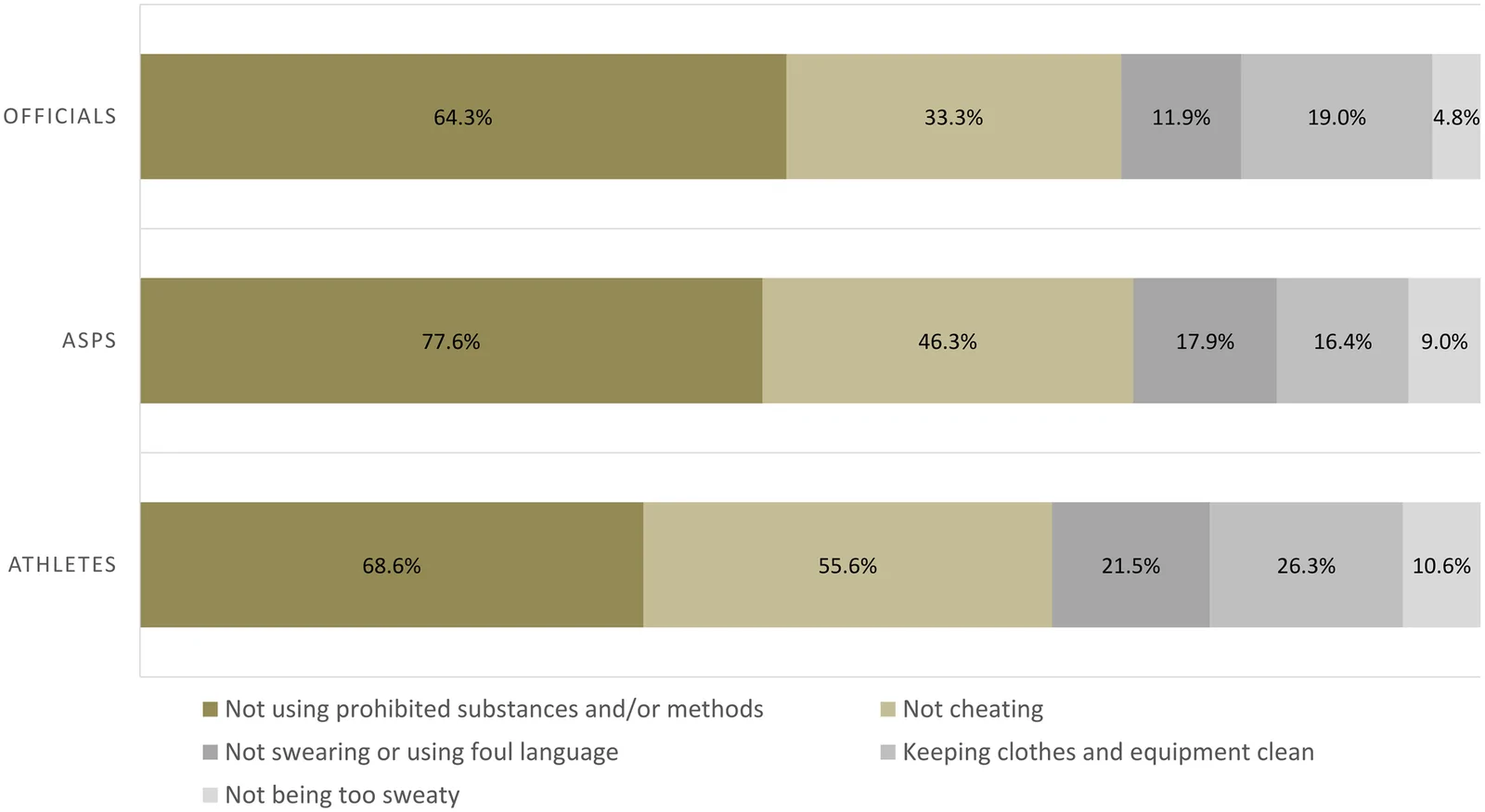
Endorsement of alternative definitions of clean sport across participant groups. Percentages represent the proportion of individuals within each participant group who selected each option.

Gender differences were observed in how athletes conceptualized clean sport. Female athletes were more likely than male athletes to define clean sport as not using prohibited substances or methods (78.1% vs. 59.6%), whereas male athletes were more likely to associate clean sport with keeping clothes and equipment clean (32.7% vs. 19.0%). Both differences remained statistically significant after Holm adjustment across the five option-specific comparisons (Supplementary File S3: Table S3.3).

### 3.5 Moral evaluations of performance-enhancing practices

Moral evaluations of doping revealed clear heterogeneity. While 69.2% of participants considered the deliberate use of prohibited substances to improve performance morally unacceptable under any circumstances, 30.8% expressed either conditional acceptance or full moral permissibility of such behavior (Table 3). This distribution highlights the presence of situated moral reasoning rather than universal moral consensus.

**Table 3:** Moral evaluations of deliberate use of prohibited substances across participant groups.

Conditions	Athlete	ASP	Other	Total
Deliberately using prohibited substances to improve performance is morally acceptable under any circumstances.	54	9	5	68
Deliberately using prohibited substances to improve performance is morally acceptable under some circumstances, but wrong in others.	45	4	7	56
Deliberately using prohibited substances to improve performance is morally unacceptable under any circumstances.	194	54	30	278
Total	293	67	42	402

Presented as frequency counts.

At the same time, these moral positions did not differ significantly by role. A chi-square test showed no statistically significant association between participant group and moral stance on deliberate use of prohibited substances, *χ*^2^(4) = 6.87, *p* = .143 (Fisher–Freeman–Halton exact *p* = .136), suggesting that athletes, ASP, and officials showed broadly similar patterns in their moral evaluations.

Despite the gender difference in the importance attached to clean sport, female and male athletes showed similar moral evaluations of deliberately using prohibited substances. Gender was not significantly associated with moral position, *χ*^2^(2) = 1.84, *p* = .398 (see Supplementary File S3: Table S3.4).

Further nuance emerged when participants were asked which forms of performance-enhancement they considered morally acceptable. Legal and medically justified practices, such as supplements free of prohibited substances (52.0%) and medication for genuine medical conditions (30.6%), were widely accepted. By contrast, overtly prohibited practices were endorsed by smaller, but non-negligible, minorities, particularly when framed around recovery or training rather than competition. These distinctions illustrate that moral boundaries around enhancement were context dependent and negotiated rather than strictly rule based.

The distribution of responses across acceptable enhancement practices appeared to vary somewhat between athletes, ASP, and officials (Figure 4), statistically significant differences were not detected. Because participants could select more than one option, each performance-enhancement practice was recoded as a separate binary variable indicating whether the participant selected or did not select the option. Endorsement of each option was then compared across athletes, athlete support personnel, and officials using separate 3 × 2 contingency-table tests. No statistically significant role-based differences were found for any option, with all Holm-adjusted *p*-values exceeding.05 and all effect sizes being small (Cramer's *V* = .013 to.119). The largest descriptive difference concerned over-the-counter, non-prohibited medication, which was endorsed by 22.2% of athletes, 34.3% of ASP, and 16.7% of officials, although this difference was not statistically significant, *χ*^2^(2) = 5.72, *p* = .057, Holm-adjusted *p* = .459, Cramer's *V* = .119. Overall, the findings indicate broadly similar patterns of endorsement across participant roles. See Supplementary File S3: Table S3.5.

**Figure 4:**
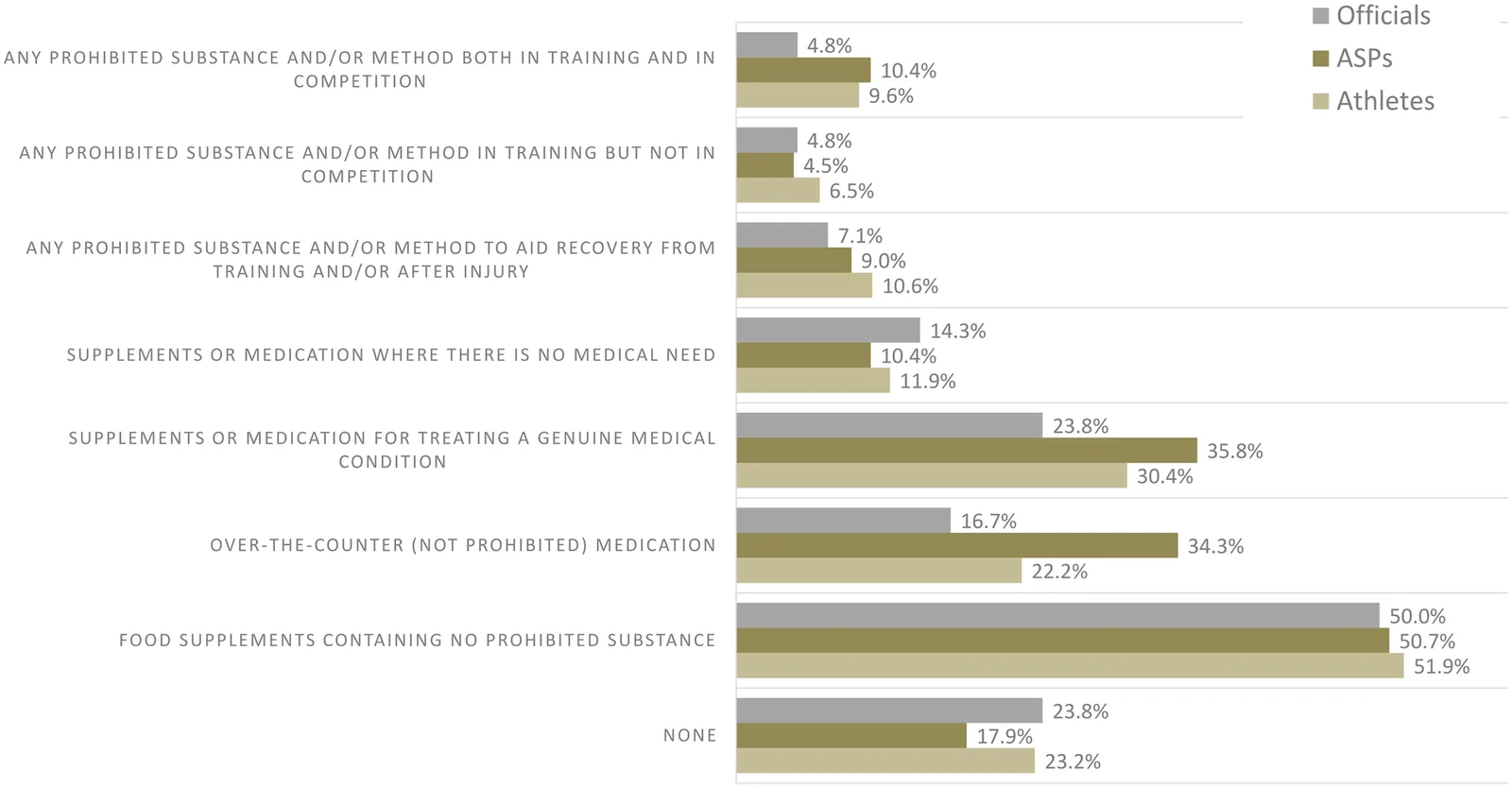
Relative endorsement of morally acceptable performance-enhancing practices across participant groups. Percentages represent the proportion of individuals within each participant group who selected each option.

Collectively, the findings suggest that moral nuance in relation to enhancement was evident across all groups, with no strong evidence of systematically different moral frameworks by participant role.

Female and male athletes showed broadly similar patterns in the enhancement practices they considered morally acceptable. Although female athletes more frequently endorsed supplements or medication for treating a genuine medical condition than male athletes (36.5% vs. 25.0%), this difference did not remain statistically significant after adjustment for the eight option-specific comparisons (Supplementary File S3: Table S3.6).

### 3.6 Perceptions of anti-doping legitimacy

Participants expressed strong support for the current anti-doping system across all four legitimacy indicators. As shown in Figure 5, mean scores were consistently high across groups for the view that current anti-doping rules are fully justified because they protect clean sport, are effective in protecting clean sport, are implemented equally across sports and countries, and are fair to all athletes. Across the full sample, item means ranged narrowly from 1.54 to 1.60, with a median of 2.00 for each indicator, indicating generally strong endorsement of both normative and procedural legitimacy.

**Figure 5:**
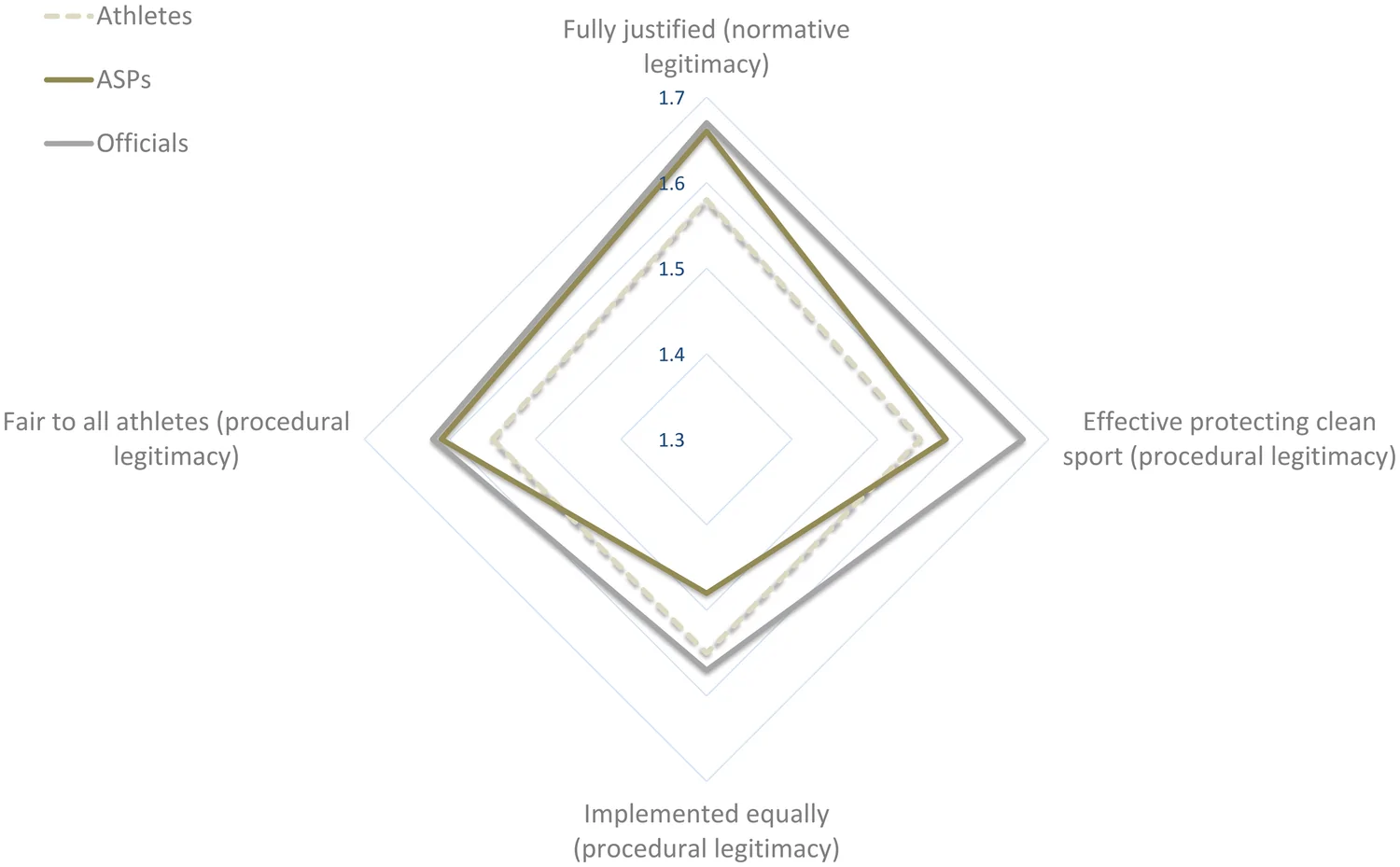
Perceptions of anti-doping legitimacy across participant groups.

Although Figure 5 suggests that officials and ASPs tended to report slightly higher legitimacy scores than athletes on some items, these differences were not statistically significant. Kruskal–Wallis tests showed no significant between-group differences for perceptions that the anti-doping rules are fully justified, *H*(2) = 1.28, *p* = .529; effective in protecting clean sport, *H*(2) = 1.79, *p* = .408; implemented equally across sports and countries, *H*(2) = 0.17, *p* = .918; or fair to all athletes, *H*(2) = 0.46, *p* = .795. There was also no significant difference across the four legitimacy indicators themselves, as indicated by the Friedman test, *χ*^2^(3) = 4.85, *p* = .183, suggesting that support was distributed relatively evenly across normative legitimacy (“doing the right thing”) and procedural legitimacy (“doing it well”).

At the same time, the distributions were not entirely uniform. Consistent with the descriptive pattern, a small minority expressed neutrality or disagreement, indicating that legitimacy was broadly endorsed but not absolute. The observed pattern is in line with legitimacy theory, which distinguishes general acceptance of an institution's right to exercise authority from evaluations of particular procedures, practices, or outcomes; broad support for a regulatory system can therefore coexist with criticism of specific aspects of its implementation (31–33). In the present case, this pattern emerged within a geographically, institutionally developmentally, and potentially culturally diverse Games population, suggesting that strong legitimacy perceptions can coexist with moral plurality when anti-doping engagement is experienced as respectful, meaningful, and supportive.

Female athletes recorded descriptively higher ratings than male athletes across all four anti-doping legitimacy indicators. However, none of the gender differences remained statistically significant after Holm adjustment, indicating broadly similar levels of perceived legitimacy among female and male athletes (Supplementary File S3: Table S3.7).

Correlation analysis further indicated that the importance attached to clean sport was positively associated with all four legitimacy indicators, although these associations were modest in size (Spearman's *r* = .224 to.273, all *p* < .001). This suggests that participants who more strongly valued clean sport were somewhat more likely to endorse the anti-doping system as morally justified and procedurally fair, and vice versa (Table 4). In contrast, the legitimacy items were very strongly intercorrelated (Spearman's *r* = .835 to.908, all *p* < .001), indicating that normative legitimacy and procedural legitimacy were experienced as closely connected rather than distinct dimensions in this sample. Together, these results suggest that support for clean sport provides part of the basis for legitimacy perceptions, but that evaluations of the anti-doping system itself form a particularly coherent and mutually reinforcing set of beliefs.

**Table 4:** Associations between importance of clean sport and anti-doping legitimacy perceptions.

Values in action measures	Normative legitimacy (just)	Procedural legitimacy: effective (appropriate)	Procedural legitimacy (fair process)	Procedural legitimacy (fair outcome)
Importance of clean sport	.273**	.259**	.229**	.224**
Normative legitimacy: The current anti-doping rules are fully justified because they protect clean sport	1.000	.867**	.835**	.840**
Procedural legitimacy: The current anti-doping rules are effective in protecting clean sport		1.000	.899**	.908**
Procedural legitimacy: The current anti-doping rules are implemented equally in all sports and all countries			1.000	.864**
Procedural legitimacy: The current anti-doping rules are fair to all athletes				1.000

** Correlation is significant at the 0.01 level (2-tailed).

The four legitimacy indicators were strongly intercorrelated among both female and male athletes. The associations of perceived effectiveness with equal implementation and fairness were stronger among male athletes than female athletes, and both differences remained significant after Holm adjustment; the other gender differences in correlation magnitude were not statistically significant (Supplementary File S3: Table S3.8). The result does not indicate that legitimacy was more strongly endorsed by male athletes. Rather, it indicates that male athletes' responses to the effectiveness, equality, and fairness indicators were more tightly aligned but legitimacy perceptions formed a highly coherent pattern for both genders.

### 3.7 Qualitative feedback and recommendations

Feedback from the open-ended question supports several practice-relevant enhancements, which can be usefully categorized as programmatic and educational improvements. Importantly, these suggestions were framed as opportunities to expand and enrich an already well-received outreach program, rather than to correct perceived shortcomings.

Programmatic enhancements focused primarily on engagement and format. Participants suggested expanding the range of interactive activities, games, and mini-games, increasing incentives or small giveaways, and offering more varied or meaningful activities across the duration of the event. A small number of responses also highlighted the value of greater opportunities for interaction and exchange, including informal dialogue and international exchange within the outreach space. These suggestions reinforce the importance of enjoyment and accessibility in major-games settings, where athletes operate within a distinctive “Games bubble” and may be more receptive to outreach that is engaging and playful rather than cognitively demanding.

Educational enhancements emphasized continuity and practical relevance. Participants recommended extending clean sport and anti-doping education, raising awareness through ongoing messaging, and providing practical resources, such as a pocket-sized clean sport guide. These suggestions align closely with the study's values-in-action framework, underscoring the need for education that supports moral reflection and everyday decision-making rather than abstract rule recitation.

## 4 Discussion

This study demonstrates that major multisport events can function as effective platforms for large-scale studies collecting geographically and institutionally diverse data drawn from Games participants when research is embedded within athlete outreach activities. Outreach programs at the Olympic and Paralympic Games, Continental Games, and other regional multisport competitions have long been recognized as valuable mechanisms for athlete education and dialogue (19, 22), and increasingly as opportunities for empirical research (e.g., 12–14). However, these educational and research functions have rarely been conceptualized as an integrated enterprise in which each can support and enhance the other. The substantive findings reported here therefore should be viewed as a secondary benefit of the “research in the games bubble” approach. The primary contribution of the study is demonstrating that geographically and potentially culturally diverse data collection can be embedded within major-event outreach programs in a manner that is feasible, acceptable to participants, and compatible with a positive game experience.

### 4.1 Outreach evaluation

A central finding of the present study was that the outreach-embedded approach was not only feasible, but also positively experienced across participant groups. Satisfaction scores across program content, activity format, and staff engagement were consistently high, with most respondents reporting that the outreach was enjoyable, informative, and supportive. Participants also indicated that visiting the outreach center increased their awareness of anti-doping issues and their anti-doping knowledge, and contributed positively to their overall Games experience. Importantly, these positive evaluations did not differ significantly across athletes, ASP, and officials. Taken together, these findings suggest that outreach at major events can serve simultaneously as an educational, relational, and research platform rather than merely as a compliance-oriented communication exercise.

The Signature & Photo Opportunity and the WADA Anti-Doping Knowledge Quiz emerged as the most highly rated activities. This finding suggests that participants were particularly attracted to activities that were either personally meaningful and memorable, such as the opportunity to make a visible commitment to clean sport and document their participation, or highly accessible, as exemplified by the multilingual WADA quiz. The popularity of these activities highlights the value of combining personal engagement with inclusive educational delivery. In contrast, the Prohibited List AR Shooting Game received comparatively lower ratings. One possible explanation is that the activity required participants to simultaneously apply anti-doping knowledge and maintain attention to task-specific rules, namely identifying prohibited substances while avoiding clean sport-related targets. This greater cognitive demand may have reduced enjoyment within a recreational game environment, where participants may have preferred more straightforward and immediately rewarding forms of engagement.

The findings also indicate that participants did not engage with outreach in a superficial or purely instrumental way. Overall program ratings and perceived increases in anti-doping awareness and knowledge were strongly positively associated. Correlations between positive program experience, reported increases in awareness, and reported increases in knowledge suggest that enjoyment, learning, and overall evaluation formed an integrated experience of the program. As these were stand-alone customer-feedback questions rather than items designed to measure distinct latent constructs, the correlations should be interpreted descriptively. Their magnitude may reflect consistency in participants' evaluations, but may also be influenced by a general positive evaluation of the outreach experience. Open-ended feedback reinforced this interpretation: rather than identifying major shortcomings, participants mainly recommended expanding interactive activities, games, opportunities for exchange, and practical educational materials. This pattern suggests that anti-doping outreach at major events may be particularly effective when delivered in ways that are engaging, accessible, and aligned with the social environment of the event itself.

Beyond feasibility, the study also makes an epistemic contribution. By embedding research in a live outreach setting at a regional major games outside Europe, the project demonstrates a practical mechanism for diversifying who is represented in anti-doping research and where such evidence is generated. This should not be understood as a substitute for deeper, locally grounded research, but rather as a strategic entry point that can help address longstanding geographical and cultural imbalances in the evidence base.

### 4.2 Clean sport, moral plurality, and anti-doping legitimacy

Across the sample, participants expressed strong endorsement of clean sport and broad support for the anti-doping system. Clean sport was rated as highly important overall, and perceptions of legitimacy were consistently strong across all four indicators examined: whether anti-doping rules were justified because they protect clean sport, effective in doing so, implemented equally across sports and countries, and fair to all athletes. At the same time, the data do not support a simplistic interpretation in which support for clean sport automatically translates into uniform moral reasoning or unquestioned compliance.

When asked what “clean sport” meant to them, participants most often associated the concept with not using prohibited substances or methods and, secondarily, with not cheating. However, response patterns differed significantly across athletes, ASP, and officials, with ASP and officials showing somewhat stronger rule-based interpretations than athletes. These findings are consistent with the view that clean sport is grounded in ethics, fair play, honesty, and respect for rules, but that these values are interpreted and prioritized somewhat differently across groups and, potentially, across cultures (25, 34–36). Such variation does not imply normative fragmentation; rather, it suggests that shared commitment to clean sport may coexist with differing emphasis in how the concept is understood and enacted.

The moral evaluation findings deepen this interpretation. Most participants regarded the deliberate use of prohibited substances to improve performance as morally unacceptable under any circumstances, but a substantial minority endorsed either conditional acceptance or full permissibility. In addition, legal supplements free of prohibited substances and medication for genuine medical conditions were accepted more readily than overtly prohibited practices. This pattern aligns with prior work in moral psychology and sport ethics showing that attitudes toward doping are shaped not only by formal rules, but also by moral identity, perceptions of fair play, social norms, and contextual considerations (29, 37). Ethical analyses further illustrate that athletes often differentiate between supplementation, prohibited doping, medically justified treatments, and technological enhancement in ways that reflect negotiated moral boundaries rather than simple compliance or deviance (6, 23).

Accordingly, the present findings support the argument that doping-related judgments are best understood as forms of context-dependent moral reasoning rather than as outcomes of informational deficits alone (24, 38). Athletes and other stakeholders do not appear simply to sort practices into a binary clean/unclean framework. Instead, they make distinctions shaped by perceived intention, fairness, medical necessity, social norms, and the status of particular enhancement practices. This is consistent with broader formulations of situated morality in sport, in which judgments emerge through context-specific interpretations of fairness, responsibility, and sport culture (6, 24, 39). From this perspective, values-based anti-doping education should not assume universal consensus around a fixed moral code; rather, it should engage with the interpretive and dialogic nature of moral judgment in high-performance sport.

The findings related to perceived legitimacy are also important. Legitimacy theory (32, 33) provides a useful framework for understanding engagement with anti-doping systems. Normative legitimacy refers to whether anti-doping is perceived as necessary and morally justified, grounded in shared values and collective goals (25). Procedural legitimacy concerns whether anti-doping rules and processes are experienced as fair, transparent, and respectful, and is shaped through direct interactions with the system and perceptions of how rules are applied in practice. In the present study, legitimacy indicators were not only high overall, but also very strongly intercorrelated, suggesting that normative and procedural legitimacy were experienced as closely linked rather than clearly separable domains. At the same time, a small minority expressed neutrality or disagreement on some aspects of anti-doping legitimacy, indicating that legitimacy was broad but not absolute. The most contested part of anti-doping legitimacy is equal implementation where ASPs recorded the mean score.

This pattern is consistent with prior legitimacy research showing that compliance and trust depend less on deterrence alone and more on perceptions of procedural fairness, transparency, and respect (25, 40). Studies of athletes' experiences with anti-doping proceedings similarly suggest that perceived injustice, lack of voice, or unequal treatment can weaken acceptance of otherwise widely endorsed rules, particularly in less-resourced or developing sport systems (41). The present findings therefore reinforce the importance of dialogue-based engagement approaches, in which athletes are invited to reflect on, question, and contribute to anti-doping practice, rather than being positioned solely as subjects of surveillance and enforcement.

The gender-related pattern among athletes suggests that clean sport may carry different salience and meanings for female and male athletes, even where their broader evaluations of anti-doping legitimacy and performance-enhancement practices remain largely similar. These exploratory findings should not be interpreted as evidence of fixed gender differences, but they highlight the value of examining how gender intersects with sporting, cultural, and institutional contexts in shaping clean-sport perspectives. Future event-based research should investigate these patterns using more nuanced measures and qualitative approaches capable of exploring the experiences and reasoning underlying them.

### 4.3 Policy and practice implications

The findings carry several implications for anti-doping education, outreach, and policy. They suggest that outreach activities at major multisport events can simultaneously support education, legitimacy building, and evidence generation, provided they are designed with bidirectional engagement in mind. In the present study, outreach was experienced as valuable, supportive, and educational, and these positive interactions likely contributed to the strong legitimacy perceptions observed across groups. This supports the view that respectful, meaningful engagement may itself function as a legitimacy-building practice within highly regulated sporting environments.

The findings also highlight the importance of designing outreach and research in ways that fit the temporal and social conditions of the “games bubble.” Major events create a distinctive environment in which athletes and their entourage may be temporarily more open to engagement, but only if the activity is perceived as worthwhile and does not impose excessive cognitive or temporal burden. In the Harbin study, the questionnaire was completed under 5 min, suggesting that brevity and accessibility were important design strengths. In this context, prioritizing short, interactive, and intuitive formats is not merely a practical compromise; it is arguably an ethically appropriate and methodologically defensible choice.

Outreach-embedded research should prioritize action-oriented and contextually meaningful measures. The present study's values-in-action framework moved beyond abstract attitudes by exploring how participants interpreted clean sport, enhancement, and anti-doping legitimacy in evaluative terms. This logic could be extended through the inclusion of forced-choice items, such as *Would You Rather…?* (WYR) scenarios, which can illuminate value trade-offs while remaining intuitive and engaging (42, 43). Policy-relevant survey designs could usefully combine WYR items with brief measures of: (a) the meaning and importance of clean sport (normative legitimacy); (b) self-assessed anti-doping literacy and efficacy, and (c) perceptions of fair process and effective outcomes (procedural legitimacy). Including athlete entourage members within these designs would further enhance practical value by producing insights relevant to the broader ecology of anti-doping decision-making.

Collecting data at regional major games outside Europe should be viewed as a strategic complement to, not a replacement for, in-depth local research. A standardized core questionnaire administered across multiple major events, supplemented by locally relevant items, could generate a valuable multi-sport, multi-national dataset while preserving flexibility and cultural sensitivity. Such an approach would allow both comparability and contextualization, helping to address epistemic imbalance without imposing epistemic uniformity. This is especially important given continuing research gaps in underrepresented regions and the growing recognition that cultural, social, and economic factors shape perceptions of doping, clean sport, and anti-doping legitimacy.

A collaborative model that extends beyond the research team to include local anti-doping educators, event organizers, and athlete clean sport ambassadors is likely to maximize both data quality and participant experience. Researchers contribute methodological expertise in study design, instrument development, data collection, and analysis, but the practical and contextual value of outreach-embedded research can be substantially strengthened when these partners are involved from the outset. Local anti-doping educators can help ensure that research content is aligned with outreach goals and communicated in ways that are accessible and contextually appropriate. Event organizers play a critical enabling role by facilitating space allocation, scheduling, and the infrastructural conditions needed for effective engagement. Athlete clean sport ambassadors, through their direct and credible interactions with participants, can further enhance trust, relevance, and willingness to engage. Designing outreach content, activities, and data collection collaboratively in this way offers the best of both worlds: research that is methodologically rigorous and outreach that is practically meaningful, participant-centered, and responsive to the realities of the event setting.

### 4.4 Methodological contribution, strengths, and limitations

A key novel contribution of this study lies in demonstrating how outreach-embedded research can function as both a methodological strategy and an epistemic intervention in anti-doping social science. Although athlete outreach has long been used to deliver education and raise awareness, its potential as a structured mechanism for generating empirically robust, geographically and potentially culturally diverse data remains underused. The present study shows that when outreach is paired with a brief and contextually appropriate questionnaire, it is possible to capture *in situ* perceptions of clean sport, enhancement, and legitimacy at the point of engagement with the anti-doping system itself. This differs meaningfully from retrospective survey work or decontextualized hypothetical judgments by situating responses within a lived event environment.

This design has several strengths. It enabled access to a geographically diverse sample that would be difficult to assemble using conventional recruitment strategies. It also enhanced ecological validity by collecting responses during a real anti-doping outreach encounter rather than in an artificial research setting. In addition, the approach appears to have been well tolerated by participants, as reflected in short completion times and high satisfaction with the outreach environment overall.

At the same time, several limitations should be acknowledged. First, participants were recruited from individuals who chose to engage with the Athlete Engagement & Education Centre and complete the embedded survey. The sample therefore may overrepresent individuals with greater interest in anti-doping, more positive attitudes toward outreach activities, or greater willingness to engage with anti-doping personnel, or motivated by the opportunity to receive a souvenir. In addition, because the survey was completed within the outreach environment and recruitment was facilitated by Clean Sport Ambassadors, responses may have been influenced by social desirability, courtesy, or situational response biases. Consequently, the findings should not be interpreted as representative of all Games participants. Data collection was entirely electronic and required access to a suitable mobile device, together with either personal mobile data or the local internet connection available at the venue. Although venue internet access may have reduced this barrier, the electronic-only format may still have discouraged or excluded individuals without a suitable device or sufficient digital familiarity.

Second, the questionnaire was available only in English and Chinese, reflecting the established language versions available for use in the host-event setting. This may have created comprehension barriers or discouraged participation among individuals who were not sufficiently proficient in either language. Moreover, the language version selected by each participant was not retained, preventing examination of possible language-related differences in responses. Representation from 28 countries and regions should therefore be understood as geographical and contextual diversity within the outreach sample, rather than as evidence of linguistic representativeness or cross-cultural measurement equivalence. Future event-based studies should provide additional language versions aligned with the participating populations, record the language of completion, and examine whether response patterns vary across language groups. Furthermore, the data were self-reported and cross-sectional, which limits conclusions about actual behavior or longer-term attitudinal change. Third, although the sample was comparatively large and diverse for an event-based study, some subgroup analyses (particularly those involving multiple categories of acceptable enhancement practices) were affected by sparse cell counts and should be interpreted with caution.

The program rating and perceived awareness and knowledge questions were brief, stand-alone customer-feedback style items. Although these were not intended as components of a validated psychometric scale, the strong correlations among these evaluations may partly reflect shared method variance or a general positive halo surrounding the outreach experience. The awareness and knowledge items assessed participants' perceived educational benefit rather than objectively measured learning. The findings should therefore not be interpreted as demonstrating distinct but integrated dimensions of program effectiveness.

At the same time, the study was designed primarily to evaluate the feasibility and acceptability of outreach-embedded research rather than population attitudes. From this perspective, the high participation volume, short completion times, and consistently positive program evaluations suggest that integrating brief research activities into athlete outreach was operationally feasible and did not appear to detract from participants' overall outreach experience. A limiting factor is that the very features that make outreach-embedded research feasible in major event settings (e.g., brevity, accessibility, and low participant burden) inevitably limit measurement depth. This trade-off should not be viewed as a weakness alone, but as a design consequence of conducting research ethically and effectively in a high-demand sporting environment.

### 4.5 Future directions

Future research can build on this study in several ways. Replication across other regional and global multisport events would help determine whether the positive reception of outreach and the patterns of moral and legitimacy judgments observed here are specific to Harbin 2025 or more broadly generalizable. Comparative work across sports, regions, and levels of anti-doping system development could also clarify how meanings of clean sport and perceptions of anti-doping legitimacy vary across institutional and cultural contexts. The greater importance attached to clean sport among female athletes, together with gender differences in how clean sport was conceptualized and tentative differences in the acceptability of some practices, warrants further investigation.

Alongside the incorporation of newer methodological approaches, such as WYR-type questions (42, 43), modest refinements to existing questionnaire items could generate more nuanced and analytically useful data. For example, current measures that combine the use of prohibited substances for recovery into a single, broad item could be disaggregated to distinguish between recovery from injury and recovery from training. Treating these as separate contexts may reveal meaningful differences in athletes' reasoning, perceived acceptability, and vulnerability to prohibited substance use.

A further area for refinement concerns how “clean sport” is operationalized. Rather than relying solely on abstract definitions (e.g., clean sport is being drug free vs. being cheating free), future research could examine whether athletes understand clean sport primarily through identity, for example, “Who is a clean athlete?”, or through behavioral choices, such as “What constitutes clean performance enhancement?” (6). This distinction could provide richer insight into how athletes negotiate the boundaries between acceptable and unacceptable practices. It would also align well with a values-in-action framework by shifting attention from abstract endorsement of clean sport values (e.g., 25, 35, 44, 45) to how these values are interpreted, enacted, and sometimes contested in real-world performance contexts (23).

Future outreach evaluations would benefit from a standard set of questions aligned with the core components of the International Standard for Education, including awareness, knowledge, and values-based beliefs supporting clean-sport compliance. Rather than relying solely on participants' perceptions that their knowledge increased, evaluation should include contextualized questions assessing whether participants can apply anti-doping information to realistic situations. Knowledge may therefore be better conceptualized as anti-doping competency or literacy, encompassing the ability to recognize relevant risks, identify appropriate sources of information, understand rules and conditions, and capable of making informed decisions in contexts such as supplement use, medication, testing, and reporting concerns. A consistent but adaptable set of measures would strengthen comparisons across events.

There is also clear scope for more qualitative follow-up work. Interviews or focus groups with athletes, ASP, and officials could illuminate how participants interpret ambiguous enhancement scenarios, how they distinguish medical treatment from performance enhancement, and how they reconcile support for anti-doping with critiques of fairness or implementation. Such work would be particularly valuable in underrepresented regions, where local moral frameworks, collective understandings of responsibility, and culturally specific notions of fair play may not map neatly onto dominant Western models. In this respect, the present study should be seen as opening space for deeper, contextually grounded enquiry rather than closing it.

## 5 Conclusion

Overall, this study demonstrates that outreach-embedded research at a major regional multisport event is feasible, acceptable, and capable of generating useful practice-relevant evidence without appearing to detract from participants' Games experience. In the distinctive “Games bubble” of the 2025 Asian Winter Games, a brief questionnaire was successfully integrated into athlete engagement activities, reaching athletes, athlete support personnel, and officials from 28 countries and regions. High satisfaction scores, positive overall programme evaluations, and strong willingness to recommend the outreach centre suggest that the embedded data collection was well tolerated and compatible with a positive outreach experience. The substantive findings, including strong support for clean sport and anti-doping legitimacy alongside nuanced moral reasoning about performance enhancement, should be interpreted as exploratory insights from those who engaged with the outreach rather than as representative evidence of all Games participants. The primary contribution of the study is therefore methodological and practical: it shows that carefully designed, low-burden research can be embedded within major-event outreach in ways that support engagement, generate geographically and potentially culturally diverse data, and create an entry point for deeper, locally grounded research. Outreach-embedded research should not replace more intensive qualitative, longitudinal, or context-specific studies, but it offers an efficient and ethically appropriate complement for broadening the anti-doping evidence base beyond the settings that have historically dominated the field. When outreach and research are designed together from the outset, they can provide the “best of both worlds”: participant-centered engagement that is meaningful in the event setting, and research that is responsive to the voices, experiences, and priorities of athletes and their entourages.

## Data Availability

The raw data supporting the conclusions of this article will be made available by the authors upon reasonable request, without undue reservation.
