# Supplementary material for: Research in the “Games Bubble”: leveraging outreach-embedded data collection at the 2025 Harbin Asian Winter Games
Source: Front Sports Act Living. 2026 Oct 7;8:1945134. doi: 10.3389/fspor.2026.1945134 (PMC13683366; doi:10.3389/fspor.2026.1945134)
Supplement: Supplementary file 1 [file Supplementaryfile1.pdf]

Supplementary Material

Supplementary file 1: Survey questions

Supplementary Table 1. Harbin 2025 outreach survey questions, scoring instructions, and constructs

	Question	Scoring	Construct
Q1	Your country/region	Single-choice nominal item:<br>– Afghanistan<br>– Bahrain<br>– Bhutan<br>– Cambodia<br>– China<br>– Chinese Taipei<br>– Democratic People's Republic of Korea<br>– Hong Kong, China<br>– India<br>– Islamic Republic of Iran<br>– Japan<br>– Kazakhstan<br>– Kyrgyzstan<br>– Lebanon<br>– Macao, China<br>– Malaysia<br>– Mongolia<br>– Other<br>– Pakistan<br>– Philippines<br>– Qatar<br>– Republic of Korea<br>– Saudi Arabia<br>– Singapore<br>– Sri Lanka<br>– Thailand<br>– Turkmenistan<br>– Uzbekistan	Country/region / participant origin
Q2	Gender	Single-choice nominal item:<br>– male<br>– female	Gender
Q3	Age	Open numeric response (years)	Age
Q4	You are an…	Single-choice nominal item:	Participant role

		– Athlete<br>– Coach<br>– Team official<br>– Medical personnel<br>– Other support personnel<br>– Other	
Q5	What is your sport and discipline?	Single-choice nominal item:<br>– Alpine Skiing<br>– Biathlon<br>– Cross-Country Skiing<br>– Curling<br>– Figure Skating<br>– Freeski Big Air<br>– Freeski Halfpipe<br>– Freestyle Aerials<br>– Ice Hockey<br>– Short Track Speed Skating<br>– Ski Mountaineering<br>– Snowboard Big Air<br>– Snowboard Halfpipe<br>– Snowboard Slopestyle<br>– Speed Skating<br>– Other	Sport / discipline
Q6	Have you had any anti-doping education before you came to Harbin 2025 Athlete Engagement & Education Centre?	Binary single-choice item: Yes / No.	Prior anti-doping education exposure
Q7	What type of education did you have?	Multiple-response items:<br>– A seminar<br>– An e-learning course<br>– Discussion with other people<br>– Reading outreach leaflets and brochures on my own<br>– Other (please specify what)<br><br>Code each option as 1 = selected, 0 = not selected. Asked of respondents indicating prior education in Q6.	Prior anti-doping education modality

Q8	Which organization provided the anti-doping education you took part in?	Multiple-response item;<br>– International Sport Federation (IF)<br>– National Anti-Doping Agency (NADO)<br>– Regional Anti-Doping Agency (RADO)<br>– World Anti-Doping Agency (WADA)<br>– I don't remember<br><br>Code each provider option as 1 = selected, 0 = not selected. Asked of respondents indicating prior education in Q6.	Provider/source of prior anti-doping education
Q9	Why did you visit the Harbin 2025 Athlete Engagement & Education Centre?	Multiple-response item:<br>– I thought it looked interesting as I walked past so I stopped for a visit<br>– I wanted to take part in the activities and win prizes<br>– I wanted more information on anti-doping<br>– I was recommended to go by my support team<br>– I was recommended to go by a friend/teammate<br>– I was required by my team<br>– I've been to a similar booth in major event before and I wanted to visit again<br>Code each reason as 1 = selected, 0 = not selected.	Motivations for outreach engagement
Q10	Satisfaction with content<br>Satisfaction with activity form<br>Satisfaction with service from staff and volunteers	5-point rating item anchored by 1 = unsatisfied and 5 = very satisfied; intermediate values (2–4) preserve increasing satisfaction.	Program satisfaction
Q11	What's your favourite activity in the Harbin 2025 Athlete Engagement Program?	Multiple-response item (up to 3 selections): See supplementary file 2	Preferred outreach activities

Q12a	Visiting the Harbin 2025 Athlete Engagement & Education Centre contributed actively to my Harbin 2025 experience	4-point agreement item: 1 = strongly disagree, 2 = disagree, 3 = agree, 4 = strongly agree. Recoded as -2, -1, +1, +2.	Perceived contribution to Games experience
Q12b	Visiting the Harbin 2025 Athlete Engagement & Education Centre increased my awareness of anti-doping issues	4-point agreement item: 1 = strongly disagree, 2 = disagree, 3 = agree, 4 = strongly agree. Recoded as -2, -1, +1, +2.	Perceived anti-doping awareness gain
Q12c	Visiting the Harbin 2025 Athlete Engagement & Education Centre increased my knowledge of anti-doping	4-point agreement item: 1 = strongly disagree, 2 = disagree, 3 = agree, 4 = strongly agree. Recoded as -2, -1, +1, +2.	Perceived anti-doping knowledge gain
Q13	Please give an overall score of the Harbin 2025 Athlete Engagement Program.	Slider item scored 1–10; higher scores indicate a more positive overall program evaluation.	Overall program evaluation
Q14	How likely would you recommend that a friend visit the Harbin 2025 Athlete Engagement & Education Centre?	5-point ordinal item: 1 = extremely unlikely, 2 = unlikely, 3 = neutral, 4 = likely, 5 = extremely likely.	Recommendation intention / advocacy
Q15	What does the phrase ‘clean sport’ mean to you?	Multiple-response item:<br>– Keeping clothes and equipment clean<br>– Not being too sweaty<br>– Not swearing or using foul language<br>– Not cheating<br>– Not using prohibited substances and/or methods<br>Code each definition option as 1 = selected, 0 = not selected.	Meaning/definition of clean sport
Q16	How important is clean sport to you?	5-point importance item: 1 = not important at all, 2 = not very important, 3 = neither important nor unimportant, 4 = important, 5 = very important. Recode as -2 to +2.	Importance attached to clean sport
Q17	Which of the following statements best describes your own personal feelings about deliberately using prohibited substances?	3-category single-choice item:<br>– I believe deliberately using prohibited substances to improve performance is morally acceptable under any circumstances.	Moral stance toward deliberate use of prohibited substances

		– I believe deliberately using prohibited substances to improve performance is morally acceptable under some circumstances, but wrong in others.<br>– I believe deliberately using prohibited substances to improve performance is morally unacceptable under any circumstances.	
Q18	In your opinion, what is morally acceptable for athletes to use to improve their performance in competitive sport?	Multiple-response item:<br>– Use of any prohibited substance and/or method both in training and in competition<br>– Use of food supplements containing no prohibited substance<br>– Use of any prohibited substance and/or method to aid recovery from training and/or after injury<br>– Use of over-the-counter (not prohibited) medication<br>– Use of supplements or medication for treating a genuine medical condition<br>– Use of supplements or medication where there is no medical need<br>– Use of any prohibited substance and/or method in training but not in competition<br>– None	Moral acceptability of performance-enhancing practices
Q19a	The current anti-doping rules are fully justified because they protect clean sport	5-point agreement item: 1 = strongly disagree, 2 = disagree, 3 = neither agree nor disagree, 4 = agree, 5 = strongly agree. Recoded as -2 to +2.	Normative legitimacy of anti-doping
Q19b	The current anti-doping rules are effective in protecting clean sport	5-point agreement item: 1 = strongly disagree, 2 = disagree, 3 = neither agree nor disagree,	Procedural legitimacy –

		4 = agree, 5 = strongly agree. Recode as -2 to +2.	perceived effectiveness
Q19c	The current anti-doping rules are implemented equally in all sports and all countries	5-point agreement item: 1 = strongly disagree, 2 = disagree, 3 = neither agree nor disagree, 4 = agree, 5 = strongly agree. Recode as -2 to +2.	Procedural legitimacy – equality of implementation
Q19d	The current anti-doping rules are fair to all athletes	5-point agreement item: 1 = strongly disagree, 2 = disagree, 3 = neither agree nor disagree, 4 = agree, 5 = strongly agree. Recode as -2 to +2.	Procedural legitimacy – fairness
