# Supplementary material for: Research in the “Games Bubble”: leveraging outreach-embedded data collection at the 2025 Harbin Asian Winter Games
Source: Front Sports Act Living. 2026 Oct 7;8:1945134. doi: 10.3389/fspor.2026.1945134 (PMC13683366; doi:10.3389/fspor.2026.1945134)
Supplement: Supplementary file 2 [file Supplementaryfile2.pdf]

Supplementary Material

Supplementary File 2: Brief description of the athlete engagement activities offered in 2025 in Harbin

The Athlete Engagement & Education Centre operated dual physical venues for anti-doping outreach during the 2025 Harbin Asian Winter Games, including an ice sports base (Huaqi Hotel Athletes' Village, 130 m²) and a snow sports base (Yabuli Yawangsi Hotel Zone C, 130 m²). All interactive and educational modules were co-designed by the Chinese Anti-Doping Agency (CHINADA) and the Olympic Council of Asia (OCA) under the Guarding the Games framework, adopting a values-based, gamified educational logic aligned with WADA'se International Standard for Education (ISE). The program served approximately 1,600 athletes and athlete support personnel across both venues.

The outreach activities were structured around an interactive experience flow comprising five engagement zones:

Signature & Photo Area: Participants signed a commitment wall and took photographs with themed backdrops and pull-up banners featuring CHINADA's anti-doping mascot "Ling Ling" and the Athlete Engagement Program.

Education Exhibition Area: An educational display featuring the hollowed-out Sports Spirit Wall, the Prohibited List, the 11 types of Anti-Doping Rule Violations (ADRV), doping control procedures, and sample collection equipment. This zone provided accessible, values-based education on clean sport.

Quiz Area: Utilising the WADA knowledge quiz game, participants tested their understanding of anti-doping rules and clean sport values in an interactive format.

Interactive Games Area: A series of gamified learning activities including:

- ***Anti-doping AR Shooting Game:*** Using augmented reality technology, participants shot at virtual representations of prohibited substances while avoiding shooting at clean medals
- ***Snow Run Parkour Motion Sensing Game***: A motion-sensing interactive game where players avoided items that could lead to doping violations while collecting clean medals
- ***"Ring Toss to the Spirit of Sport":*** Participants tossed rings onto clouds representing the values of the spirit of sport
- ***Frisbee Throwing Game:*** Players used frisbees to hit targets corresponding to prohibited substances and methods

Message Leaving & Prize Area: A message board for participants to leave their clean sport commitments, with souvenir items (multi-layer color stamps, postcards, canvas bags, themed stickers, pins, shaker keychains, and magnets) distributed upon completion of activities.

Staffing: The program was delivered by trained Clean Sport Ambassadors who guided participants through the experience flow, facilitated dialogue on clean sport values, and administered the outreach-embedded survey. The design integrated education, engagement, and research in a mutually reinforcing model aligned with the ISE values-based approach.
