# Supplementary material for: Research in the “Games Bubble”: leveraging outreach-embedded data collection at the 2025 Harbin Asian Winter Games
Source: Front Sports Act Living. 2026 Oct 7;8:1945134. doi: 10.3389/fspor.2026.1945134 (PMC13683366; doi:10.3389/fspor.2026.1945134)
Supplement: Supplementary file 3 [file Supplementaryfile3.pdf]

Supplementary Material

Supplementary file 3: Detailed statistical analyses

Table S3.1: Gender differences in reported importance of clean sport

Participant role	Male n	Male mean rank	Female n	Female mean rank	Mann–Whitney U	Z	Exact p, two-tailed	Holm-adjusted p	Effect size, r
Athletes	156	130.60	137	165.68	8,127.00	−4.463	<.001	<.001	.261
ASP	35	34.26	32	33.72	551.00	−0.182	.868	.868	.022
Officials	22	18.70	20	24.58	158.50	−2.263	.029	.058	.349

Notes: Importance was rated on a five-point scale recoded from −2 to +2, with higher scores indicating greater importance attached to clean sport. Exact two-tailed probabilities are reported. ASP = athlete support personnel. Holm adjustment was applied across the three gender comparisons. Effect size was calculated as $r = | Z | / \sqrt{N}$.

Table S3.2: Meaning of clean, group comparison

Clean sport meaning option	Athletes selected n (%)	ASP selected n (%)	Officials/Other selected n (%)	$\chi^2(2)$	p	Holm-adjusted p	Cramer's V
Keeping clothes and equipment clean	77 (26.3%; 95% CI 21.6-31.6)	11 (16.4%; 95% CI 9.4-27.1)	8 (19.0%; 95% CI 10.0-33.3)	3.52	.172	.688	.094
Not being too sweaty	31 (10.6%; 95% CI 7.6-14.6)	6 (9.0%; 95% CI 4.2-18.2)	2 (4.8%; 95% CI 1.3-15.8)	1.47	.479	.765	.060
Not swearing or using foul language	63 (21.5%; 95% CI 17.2-26.6)	12 (17.9%; 95% CI 10.6-28.7)	5 (11.9%; 95% CI 5.2-25.0)	2.32	.313	.765	.076
Not cheating	163 (55.6%; 95% CI 49.9-61.2)	31 (46.3%; 95% CI 34.9-58.1)	14 (33.3%; 95% CI 21.0-48.4)	8.28	.016	.080	.144
Not using prohibited substances and/or methods	201 (68.6%; 95% CI 63.1-73.6)	52 (77.6%; 95% CI 66.3-85.9)	27 (64.3%; 95% CI 49.2-77.0)	2.73	.255	.765	.082

Notes: Participants, rather than votes, are the unit of analysis. Because multiple responses were allowed, we compared whether the proportion selecting that option differs across the three participant groups for each response option separately. For "Not being too sweaty," the minimum expected count was slightly below 5. Because the Pearson chi-square result is clearly non-significant, this does not affect the substantive interpretation. Percentages are accompanied by 95% Wilson confidence intervals.

Table S3.3: Gender differences in meanings attributed to clean sport among athletes

Meaning of clean sport	Female athletes, n (%)	Male athletes, n (%)	$\chi^2(1)$	p	Holm-adjusted p	φ
Keeping clothes and equipment clean	26 (19.0%; 95% CI 13.3-26.4)	51 (32.7%; 95% CI 25.8-40.4)	7.08	.008	.031	.156
Not being too sweaty	9 (6.6%; 95% CI 3.5-12.0)	22 (14.1%; 95% CI 9.5-20.4)	4.38	.037	.109	.122
Not swearing or using foul language	34 (24.8%; 95% CI 18.3-32.7)	30 (19.2%; 95% CI 13.8-26.1)	1.33	.248	.248	.067
Not cheating	83 (60.6%; 95% CI 52.2-68.4)	79 (50.6%; 95% CI 42.9-58.4)	2.92	.088	.175	.100
Not using prohibited substances and/or methods	107 (78.1%; 95% CI 70.5-84.2)	93 (59.6%; 95% CI 51.8-67.0)	11.51	<.001	.004	.198

Note: Participants could select more than one response option. Each option was therefore recoded as a separate binary variable, selected versus not selected, and analysed using a 2 × 2 Pearson chi-square test. Percentages were calculated within gender. Holm adjustment was applied across the five option-specific comparisons. Female athletes: n=137; male athletes: n=156; total athlete sample: N=293. The phi coefficient, ϕ, is reported as the effect-size estimate. After correction, two gender differences remained significant: (1) Not using prohibited substances and/or methods which was endorsed by 78.1% of female athletes and 59.6% of male athletes, adjusted p=.004, ϕ=.198, and (2) Keeping clothes and equipment clean: endorsed by 19.0% of female athletes and 32.7% of male athletes, adjusted p=.031, ϕ=.156. The unadjusted difference for "not being too sweaty" did not remain significant after Holm adjustment. The patterns for "not cheating" and "not swearing or using foul language" also did not differ statistically by gender. Percentages are accompanied by 95% Wilson confidence intervals.

Table S3.4. Gender differences in moral evaluations of deliberate prohibited-substance use among athletes

**Moral evaluation**	**Male athletes, n (%)**	**Female athletes, n (%)**	**Total, n (%)**
Morally acceptable under any circumstances	29 (18.6%; 95% CI 13.3-25.4)	25 (18.2%; 95% CI 12.7-25.6)	54 (18.4%; 95% CI 14.4-23.3)
Morally acceptable under some circumstances, but wrong in others	28 (17.9%; 95% CI 12.7-24.7)	17 (12.4%; 95% CI 7.9-19.0)	45 (15.4%; 95% CI 11.7-19.9)
Morally unacceptable under any circumstances	99 (63.5%; 95% CI 55.7-70.6)	95 (69.3%; 95% CI 61.2-76.4)	194 (66.2%; 95% CI 60.6-71.4)
**Total**	156 (100%)	137 (100%)	293 (100%)

Notes: Counts were taken from the crosstabulation restricted to athletes. Percentages are calculated within gender and are accompanied by 95% Wilson confidence intervals. The association between gender and moral evaluation was not statistically significant, chi-square(2) = 1.84, p = .398, Cramer's V = .079. All expected cell frequencies exceeded five.

Table S3.5: Acceptability of approached to performance enhancement, group comparison

Acceptability option	Athletes n (%)	ASP n (%)	Officials n (%)	$\chi^2(2)$	p	Holm-adjusted p	Cramer's V
Food supplements containing no prohibited substance	152 (51.9%; 95% CI 46.2-57.5)	34 (50.7%; 95% CI 39.1-62.3)	21 (50.0%; 95% CI 35.5-64.5)	0.07	.966	1.000	.013
Over-the-counter non-prohibited medication	65 (22.2%; 95% CI 17.8-27.3)	23 (34.3%; 95% CI 24.1-46.3)	7 (16.7%; 95% CI 8.3-30.6)	5.72	.057	.459	.119
Supplements/medication for a genuine medical condition	89 (30.4%; 95% CI 25.4-35.9)	24 (35.8%; 95% CI 25.4-47.8)	10 (23.8%; 95% CI 13.5-38.5)	1.78	.411	1.000	.067
Supplements/medication where there is no medical need	35 (11.9%; 95% CI 8.7-16.2)	7 (10.4%; 95% CI 5.2-20.0)	6 (14.3%; 95% CI 6.7-27.8)	0.36	.835	1.000	.030
Prohibited substance/method for recovery from training or injury	31 (10.6%; 95% CI 7.6-14.6)	6 (9.0%; 95% CI 4.2-18.2)	3 (7.1%; 95% CI 2.5-19.0)	0.57	.751	1.000	.038
Prohibited substance/method in training but not competition	19 (6.5%; 95% CI 4.2-9.9)	3 (4.5%; 95% CI 1.5-12.4)	2 (4.8%; 95% CI 1.3-15.8)	0.51	.774	1.000	.036
Prohibited substance/method in training and competition	28 (9.6%; 95% CI 6.7-13.5)	7 (10.4%; 95% CI 5.2-20.0)	2 (4.8%; 95% CI 1.3-15.8)	1.16	.560	1.000	.054

None	68 (23.2%; 95% CI 18.7-28.4)	12 (17.9%; 95% CI 10.6-28.7)	10 (23.8%; 95% CI 13.5-38.5)	0.94	.627	1.000	.048

Notes: The exact question were: In your opinion, what is morally acceptable for athletes to use to improve their performance in competitive sport? Participants, rather than votes, are the unit of analysis. Because multiple responses were allowed, we compared whether the proportion selecting that option differs across the three participant groups for each response option separately. Three options had expected frequencies below five: prohibited substances/methods for recovery: minimum expected count = 4.18; prohibited substances/methods in training and competition: minimum expected count = 3.87; prohibited substances/methods in training only: minimum expected count = 2.51. Given that the observed group differences are very small and the Pearson p-values are well above .05, exact testing is unlikely to change the substantive interpretation. Percentages are accompanied by 95% Wilson confidence intervals.

Table S3.6: Gender differences in the moral acceptability of performance-enhancement practices among athletes

Performance-enhancement practice	Female athletes, n (%)	Male athletes, n (%)	$\chi^2(1)$	p	Holm-adjusted p	φ
Prohibited substance or method to aid recovery from training or injury	15 (10.9%; 95% CI 6.7-17.3)	16 (10.3%; 95% CI 6.4-16.0)	0.04	.848	1.000	.011
Prohibited substance or method in training and competition	17 (12.4%; 95% CI 7.9-19.0)	11 (7.1%; 95% CI 4.0-12.2)	2.42	.120	.718	.091
Prohibited substance or method in training but not competition	10 (7.3%; 95% CI 4.0-12.9)	9 (5.8%; 95% CI 3.1-10.6)	0.28	.596	1.000	.031
Food supplements containing no prohibited substance	72 (52.6%; 95% CI 44.2-60.7)	80 (51.3%; 95% CI 43.5-59.0)	0.05	.828	1.000	.013
Over-the-counter, non-prohibited medication	37 (27.0%; 95% CI 20.3-35.0)	28 (17.9%; 95% CI 12.7-24.7)	3.47	.063	.438	.109
Supplements or medication for treating a genuine medical condition	50 (36.5%; 95% CI 28.9-44.8)	39 (25.0%; 95% CI 18.9-32.3)	4.56	.033	.262	.125

Supplements or medication where there is no medical need	16 (11.7%; 95% CI 7.3-18.1)	19 (12.2%; 95% CI 7.9-18.2)	0.02	.895	1.000	.008
None	32 (23.4%; 95% CI 17.1-31.1)	36 (23.1%; 95% CI 17.2-30.3)	0.00	.955	1.000	.003

Notes: Participants could select more than one option. Each option was therefore recoded as a separate binary variable, selected versus not selected, and analysed using a 2 × 2 Pearson chi-square test. Percentages were calculated within gender and consequently do not sum to 100%. Holm adjustment was applied across the eight option-specific comparisons. Female athletes: $n = 137$; male athletes: $n = 156$; total athlete sample: $N = 293$. The phi coefficient, ϕ, is reported as the effect-size estimate. Before correction, female athletes were more likely to endorse supplements or medication for a genuine medical condition: Female athletes: 36.5%; Male athletes: 25.0%, $\chi^2(1) = 4.56$, $p = .033$, ϕ=.125. However, this association did not survive Holm adjustment, adjusted $p = .262$. The difference for over-the-counter non-prohibited medication also approached, but did not reach, the unadjusted significance threshold ($p = .063$, adjusted $p = .438$). All effect sizes were small.

Table S3.7: Gender comparisons anti-doping legitimacy among athletes

Anti-doping legitimacy indicator	Female athletes, M (SD)	Male athletes, M (SD)	Mann–Whitney U	Z	Exact p	Holm-adjusted p	Effect size, r
Rules are fully justified because they protect clean sport	1.69 (0.63)	1.47 (0.88)	9,504.00	−2.037	.042	.168	.119
Rules are effective in protecting clean sport	1.65 (0.64)	1.46 (0.89)	9,740.50	−1.597	.111	.222	.093
Rules are implemented equally in all sports and countries	1.67 (0.57)	1.45 (0.89)	9,602.00	−1.824	.068	.204	.107
Rules are fair to all athletes	1.61 (0.80)	1.49 (0.87)	9,914.50	−1.328	.185	.222	.078

Notes: Female athletes: $n = 137$; male athletes: $n = 156$; total $N = 293$. Items were scored from −2 to +2, with higher scores indicating stronger endorsement. Exact two-tailed p-values are reported. Holm adjustment was applied across the four legitimacy comparisons. Effect size was calculated as $r = | Z | / \sqrt{N}$. All effect sizes were small. Descriptive statistics were obtained from the gender- and role-stratified output; inferential statistics were obtained from the corresponding athlete-level gender comparisons.

Supplementary Table S3.8: Comparison of anti-doping legitimacy correlations by gender among athletes

Legitimacy indicators	Female athletes, ρ	Male athletes, ρ	z for difference	p	Holm-adjusted p
Rules justified and rules effective	0.811 (95% CI 0.745-0.862)	0.876 (95% CI 0.834-0.908)	−1.93	.054	.194
Rules justified and implemented equally	0.838 (95% CI 0.780-0.882)	0.852 (95% CI 0.802-0.890)	−0.41	.679	.679
Rules justified and fair to athletes	0.793 (95% CI 0.721-0.848)	0.865 (95% CI 0.819-0.900)	−1.97	.049	.194
Rules effective and implemented equally	0.844 (95% CI 0.788-0.886)	0.942 (95% CI 0.921-0.957)	−4.40	<.001	<.001
Rules effective and fair to athletes	0.866 (95% CI 0.817-0.903)	0.944 (95% CI 0.924-0.959)	−3.86	<.001	.001
Implemented equally and fair to athletes	0.830 (95% CI 0.769-0.876)	0.884 (95% CI 0.844-0.914)	−1.74	.082	.194

Notes: Comparing the six athlete-only Spearman correlations between female athletes (n=137) and male athletes (n=156) using Fisher's r-to-z r transformation as an approximate test of independent correlations. The pattern was broadly similar across genders, but two associations

involving perceived effectiveness were significantly stronger among male athletes. All within-gender Spearman correlations were positive and statistically significant at $p < .001$.

Differences between independent correlations were evaluated using Fisher's r-to-z transformation. Holm adjustment was applied across the six gender comparisons. Female athletes: $n = 137$;

male athletes: $n = 156$.

Because Fisher's transformation is conventionally derived for Pearson correlations, the gender-comparison tests for Spearman coefficients should be regarded as approximate and exploratory. Among male athletes, viewing anti-doping as effective was especially closely connected with viewing implementation as equal and outcomes as fair. Among female athletes, these associations were also very strong, but somewhat less pronounced. The result supports item-level reporting rather than requiring the four indicators to be treated as a psychometrically validated scale. Approximate 95% confidence intervals for each Spearman correlation were calculated using Fisher's z transformation.
